# Tailoring Discharge Summaries to Health Care Providers’ Needs (Part 1 of the Framework and Implementation of AI Tools Project): User-Centered Design Approach

**DOI:** 10.2196/80613

**Published:** 2026-03-04

**Authors:** Mieke Deschepper, Helga Rogge, Mathias Syx, Kirsten Colpaert

**Affiliations:** 1 Data Science Institute, Ghent University Hospital, Corneel Heymanslaan 10, Ghent, 9000, Belgium, +32 93321814; 2 Department of Intensive Care, Data Science Institute, Ghent University Hospital, Ghent, Belgium; 3 Department of Internal Medicine and Pediatrics, Ghent University, Ghent, Belgium

**Keywords:** large language models, clinical artificial intelligence integration, clinical AI integration, prompt engineering, discharge summaries, human-centered design, artificial intelligence, AI

## Abstract

**Background:**

Medical discharge letters are critical for continuity of care but often lack clarity and personalization, making it difficult for health care providers to retrieve essential information. While large language models (LLMs) offer potential for automating summary generation, their effectiveness depends heavily on the quality and contextual relevance of the prompts used.

**Objective:**

The objective of this study was to develop and describe a human-centered, replicable framework for creating individualized prompts that guide LLMs in generating summaries tailored to the specific needs of health care providers.

**Methods:**

A multidisciplinary workshop was conducted at Ghent University Hospital with 26 health care providers from 5 institutions, including hospitals and general practitioner networks. Participants brainstormed ideal summary formats, generating 170 ideas categorized into themes such as “structure,” “medical history,” “medication,” and “follow-up.” These insights informed the development of a 110-item structured questionnaire distributed to 33 participants. Responses were used to generate personalized and generic prompts, refined using the context, objective, style, tone, audience, and response (CO-STAR) framework.

**Results:**

“Structure and layout” (40/170, 23.5%) and “follow-up” (27/170, 15.9%) were the most emphasized categories in the workshop. The questionnaire confirmed the importance of the “follow-up” and “medical history” sections. Prompts were generated per participant and by health care type, incorporating frequently selected responses. The CO-STAR framework was applied to improve prompt clarity and alignment with clinical expectations. Communication emerged as a new category during the workshop and was universally valued in the questionnaire.

**Conclusions:**

This study presents a novel, systematic approach to prompt engineering in clinical artificial intelligence applications. By translating qualitative input into structured, individualized prompts, the framework is designed to improve the usability and relevance of artificial intelligence–generated summaries. It proposes a scalable approach for integrating human-centered design into LLM deployment in health care, with the aim of supporting more accurate, context-aware clinical documentation. However, these outcomes remain to be empirically validated; this study is limited to the design and implementation of a human-centered prompt construction pipeline in a specific multicenter setting.

## Introduction

### Background

Medical discharge letters are essential communication tools, yet their length, complexity, and variability often hinder efficient information retrieval by health care professionals, who frequently encounter difficulties in identifying key elements such as medication changes, follow-up recommendations, and significant diagnostic findings. Moreover, the informational needs of a general practitioner, a surgeon, an intensivist, and other health care providers could differ substantially.

This complexity not only hinders continuity of care but also introduces risk. In recent years, large language models (LLMs) have shown promise in helping extract, summarize, or restructure clinical documents such as medical discharge letters [1]. However, evaluating the quality and safety of such applications remains a human-centered task requiring careful planning and expert validation.

Numerous studies have evaluated the use of LLMs for generating medical discharge letters [2-4]. Despite the growing sophistication of these models, human evaluation remains indispensable for assessing the clinical accuracy, completeness, and safety of the generated outputs. Physicians, in particular, play a critical role in validating whether artificial intelligence (AI)–generated summaries address the nuanced informational needs of health care providers [2,3]. As Tam et al [5] emphasize, a structured planning phase is essential before initiating human evaluation. This phase begins with clearly defining the evaluation objectives and the specific tasks to be assessed. However, while the final evaluation is typically conducted by clinicians, the development of the prompts that guide LLMs is often carried out by engineers or a single physician. This disconnect may result in prompts that do not fully reflect the diverse and context-specific requirements of end users [2-4,6,7].

Effective medical prompt engineering is emerging as a critical skill for health care professionals navigating the integration of AI into clinical practice. As Meskó [8] emphasizes, the rise of LLMs such as ChatGPT represents a paradigm shift in health care, offering unprecedented opportunities for enhancing clinical workflows, patient communication, and decision-making. However, realizing these benefits depends heavily on the clinician’s ability to craft precise, context-aware prompts that guide AI systems toward generating accurate and relevant outputs. Patil et al [9] further highlight that well-designed prompts—grounded in medical knowledge and aligned with evidence-based guidelines—are essential for eliciting clinically meaningful responses from AI tools. They argue that prompt engineering should be recognized as a core competency in medical education, enabling physicians to harness AI effectively while maintaining ethical standards and minimizing bias. These perspectives underscore that prompt engineering is not solely a technical task. However, at present, it is neither a skill that all health care professionals possess nor one that they have time to develop in depth. Moreover, as generative AI continues to evolve, its evaluation should be approached as an ongoing process, recognizing that performance, reliability, and clinical relevance may shift over time and require continuous monitoring and adaptation. Furthermore, due to the variability in ChatGPT’s responses, evaluating its performance in health care requires repeated testing to ensure reliability. Without incorporating repetition, research findings may be biased and lack credibility [10].

In our project, Framework & Implementation of AI Tools (FRAIT), we aim to develop AI-driven solutions that are tailored to the specific needs of health care providers. The first phase of the project adopts a design thinking approach to design a prompt that accurately reflects the unique requirements and expectations of individual health care professionals. This involves close collaboration to understand their workflows and information needs and the specific clinical contexts in which they operate. Once this tailored prompt is established, the second phase will involve evaluating its effectiveness by applying it to Dutch medical discharge letters, assessing how well it extracts or summarizes relevant information. In this paper, we concentrate on the initial phase: constructing a prompt that is both meaningful and practical for the health care provider, ensuring that it aligns with their clinical and administrative objectives.

### Objectives

The aim of this study was to develop a comprehensive process that starts by identifying the needs of health care providers regarding the ideal format for medical discharge letter summaries and results in creating an individualized prompt to generate such summaries effectively.

## Methods

### Part 1: Workshop

#### Overview

Health care providers, primarily physicians, were recruited from 5 different institutions: 3 hospitals (Ghent University Hospital, AZ Oudenaarde, and AZ Sint-Lucas Ghent) and 2 general practitioner circles (Huisartsenvereniging Ghent and Huisartsenkring Schelde Leie). The target recruitment range was 20 to 30 health care providers.

The workshop took place on Wednesday, October 16, 2024, at Ghent University Hospital. The workshop followed a 3-part structure consistent with design thinking principles: an energizer to foster engagement, a brainstorming session to elicit ideas, and a feedback round to collaboratively refine and prioritize outputs.

A set of 7 questions, each with 3 possible responses (blue, yellow, and green; Table S1 in Multimedia Appendix 1) was provided to assess the level of LLM use. Results were presented as numbers and percentages. Data were collected through 2 different persons manually counting the groups and a validation based on pictures (Figure S1 in Multimedia Appendix 1).

Following an introduction to the project, the participants were organized into 6 groups, each encompassing a diverse array of institutions and specializations. We used the digital platform Padlet [11] to conduct this brainstorming session, which allowed us to document all steps in the process. The participants had the option to enter their ideas on an available laptop or through their smartphones, which did not require any log-in credentials or other personal information.

The main question for the brainstorming session was the following:

What does my ideal generated summary from a medical discharge letter look like? Eg: use of bullet points, Eg: include history. Take us through your thinking: what should be included? All ideas are welcome – personal preference counts. No consensus required, the groups are for cross-pollination / inspiration.

The emphasis was placed on both layout and content (eg, “I would like to see the accurate medical history, and I would like it to be highlighted”).

All ideas were free text, although they required a label or category. The available labels at the start of the brainstorming session were “history,” “current problems,” “medication,” “decision,” “follow-up,” “structure and layout,” and “other” or “I don’t know.” New categories could be added during the brainstorming session.

A preliminary feedback session was held after 15 minutes to discuss the label “other” in more detail for refinement. Subsequently, an additional 15-minute brainstorming session was scheduled.

#### Statistics

A word cloud was used to visualize and cluster tokens and words based on their frequency within various ideas. The number of ideas by group and category was reported as frequencies and percentages, means and SDs, and medians and IQRs.

During the workshop, results were presented by category as well as the most common words within each category. The full text of the ideas for these specific words was provided to facilitate further discussion within the group if any clarification was required.

### Part 2: Questionnaire

The ideas generated from the brainstorming session were systematically categorized. Two independent researchers (MD and HR) conducted data cleaning to eliminate duplicates and segment the ideas into smaller, manageable components. A structured table was developed (Table S2 in Multimedia Appendix 1) where each row represented a primary idea and subsequent columns contained subquestions. Each idea was assigned to a predefined category. For multiple-choice questions, the possible answers were maintained in a separate table (Table S3 in Multimedia Appendix 1). Using this structured table, a complete list of questions was generated. Additionally, we developed a prompt for each potential response in this questionnaire in Dutch, as well as an automated English translation. The questionnaire was designed using Microsoft Forms, with the embedded Microsoft Copilot used to streamline the automation process.

Initially, some general questions were added regarding the type of health care provider and the number of years in practice. An open-ended question for suggestions was included at the end. This questionnaire was distributed to all participants of the workshop and an additional group of general practitioners who will be involved in the ongoing process of the FRAIT project.

The results of the questionnaire were combined with the predetermined prompt for each answer, resulting in a unique prompt for each participant. Additionally, a generic prompt was created overall and by type of health care provider. This generic prompt selected the most frequently chosen answer for each question. For multiple-choice questions, an option must be present in at least one-third of the answers.

To transform the workshop ideas into a structured questionnaire and prompt templates, we applied a systematic multistep process. Each free-text idea was carefully reviewed and split into the smallest meaningful units, with each segment representing a single, actionable suggestion. For example, a statement such as “I want the summary to highlight medication changes and provide a clear follow-up plan” was divided into 2 distinct ideas: highlighting medication changes and providing a clear follow-up plan. Segmentation was guided by the principle that each unit should be assignable to a single category and actionable in prompt design.

Segments were then assigned to predefined categories, such as “medical history,” “medication,” “follow-up,” and “structure and layout.” If a segment fit multiple categories, it was assigned to all relevant categories (multilabel coding). New categories were created when multiple participants raised themes not covered by existing categories.

The coding process was iterative and collaborative. Two researchers independently segmented and categorized all ideas. Rather than aiming for consensus or exclusion of divergent ideas, we used a structured framework (Tables S2 and S3 and Figure S2 in Multimedia Appendix 1) to ensure that all contributions from the workshop were systematically included. MD constructed the initial framework by segmenting and categorizing all ideas, whereas HR independently reviewed the results for completeness and accuracy. Any ambiguities or uncertainties were discussed, but the primary objective was the comprehensive inclusion of all perspectives, not convergence on a single interpretation. This approach maximizes methodological transparency and reproducibility, as every idea generated during the workshop is traceable through the coding and questionnaire development pipeline.

Two Python scripts (Python Software Foundation) were created to automate data processing and prompt generation. The first script constructs a dynamic questionnaire by normalizing text, analyzing response frequencies, and reshaping data for optimal mapping between questions and answers. The second script translates completed responses into individualized and generic AI prompts using index-based mapping and neural machine translation for Dutch-to-English conversion. Both scripts aggregate and profile responses to ensure that generated prompts reflect the most common preferences among health care providers, supporting transparent and reproducible prompt engineering.

**Figure 1.**
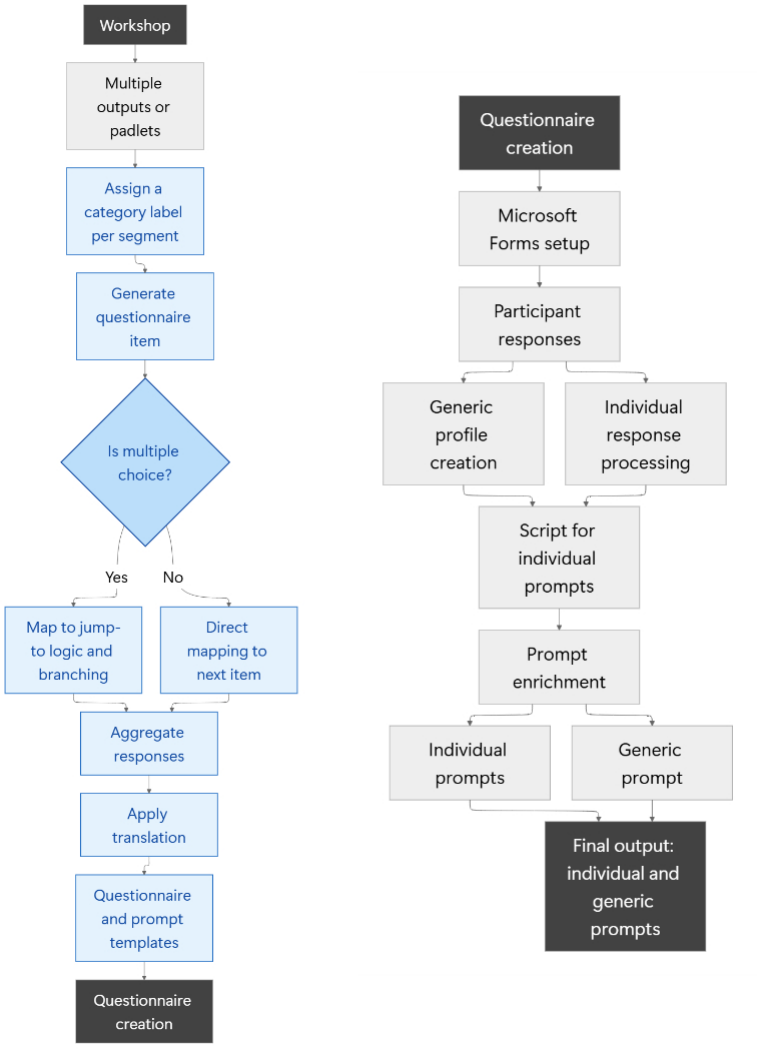
Overview of the full process flow: from multidisciplinary idea generation and categorization, through questionnaire development and automation, to the final context, objective, style, tone, audience, and response framework–optimized prompt for clinical discharge summaries.

Finally, we enhanced all prompts by incorporating segments before and after the original prompts, adhering to the context, objective, style, tone, audience, and response (CO-STAR) framework [12-14]. To operationalize the CO-STAR framework, we incorporated both participant input and established clinical communication standards during the prompt enrichment phase. Workshop and questionnaire participants were asked to articulate their preferences regarding the presentation of discharge summaries, including aspects such as clarity, formality, and professional perspective. These responses informed the initial drafting of prompts. Figure 1 illustrates the complete process.

We compared responses in the questionnaire for each category of health care provider, ensuring that there were at least 5 responses per type. Our primary emphasis was on content questions, not layout questions. Because our sample size was limited, we restricted our analysis to descriptive statistics only.

### Ethical Considerations

All approvals were granted by the ethical committee of Ghent University Hospital (ONZ-2024‐0304). The ethical committees of AZ Sint-Lucas Ghent and AZ Oudenaarde were informed of the study. Each participating organization, both hospitals and general practitioner circles, handled recruitment themselves, using their own internal communication channels and procedures to invite participants. The recruitment letter was included with our submission to the ethical committee. Informed consent was obtained from all participants before the start of the workshop (Figure S3 in Multimedia Appendix 1).

To ensure participant anonymity and data privacy, all responses collected during the workshop and questionnaire phases were anonymized before analysis. No personally identifiable information was linked to the responses, and participants were given the option to contribute via digital platforms without requiring log-in or personal credentials. Data were stored securely and accessed only by authorized research team members. These measures were implemented in accordance with institutional guidelines and approved by the relevant ethical committees.

Participants received compensation for the time they spent contributing to the project. This compensation acknowledged the substantial effort required to evaluate the summaries and attendance to the workshop, to ensure that clinicians were remunerated for their professional expertise and time commitment. No additional financial or material incentives were provided beyond this time‑based compensation.

## Results

### Part 1: Workshop

A total of 26 health care providers were recruited for this workshop (Tables S4 and S5 in Multimedia Appendix 1). However, 7.7% (2/26) of the individuals were not present during the first half hour and did not respond to the initial questions. Overall, 5 groups were formed: 4 groups consisting of 5 participants each and 1 group consisting of 6 participants.

Table S1 in Multimedia Appendix 1 lists the questions at the start of the workshop and outlines the profiles of the participants. Figure S4 in Multimedia Appendix 1 presents the outcomes of tokenizing all ideas, which are visualized using a word cloud. Figure S5 in Multimedia Appendix 1 also contains an example of the initial concepts as presented to the participants from one of the groups.

Following a 15-minute feedback session and a plenary discussion on topics categorized as “other,” a new “communication” category was established based on requests from 2 separate groups, demonstrating responsiveness to participant input. Through an iterative process of coding and review, all ideas, including those applicable to multiple categories, were systematically preserved.

At the conclusion of the workshop, a comprehensive compilation of 170 ideas generated by all groups was assembled. The complete list, along with corresponding group names, can be found in Table S6 in Multimedia Appendix 1. The “structure and layout” category contained the greatest number of ideas (40/170, 23.5%), whereas only 6.5% (11/170) of the ideas were left unlabeled by participants. “Communication,” introduced during the second round, constituted the smallest category (Table 1).

The finalized coded table served as the foundation for developing a 110-item questionnaire using branching logic for multiple-choice questions. This systematic approach facilitated the creation of both individualized and generic prompt templates.

**Table 1.** Distribution of workshop-generated ideas by category, illustrating the thematic focus and relative emphasis placed by health care providers on different aspects of discharge summary design.

Category	Participant group	Ideas (N=170), n (%)	Ideas (n), mean (SD)	Ideas (n), median (IQR)
Communication	4	8 (4.7)	2 (1.2)	2 (1-3)
Current problems	5	20 (11.8)	4 (2)	5 (3-5)
Decision	5	19 (11.1)	3.8 (1.9)	3 (3-4)
Follow-up	5	27 (15.9)	5.4 (1.1)	5 (5-6)
Medical history	5	24 (14.1)	4.8 (3.8)	4 (3-5)
Medication	5	21 (12.4)	4.2 (3.3)	3 (2-6)
Other or “I don’t know”	4	11 (6.5)	2.8 (1.5)	2 (2-3)
Structure and layout	5	40 (23.5)	8 (2.5)	8 (7-8)

### Part 2: Questionnaire

**Figure 2.**
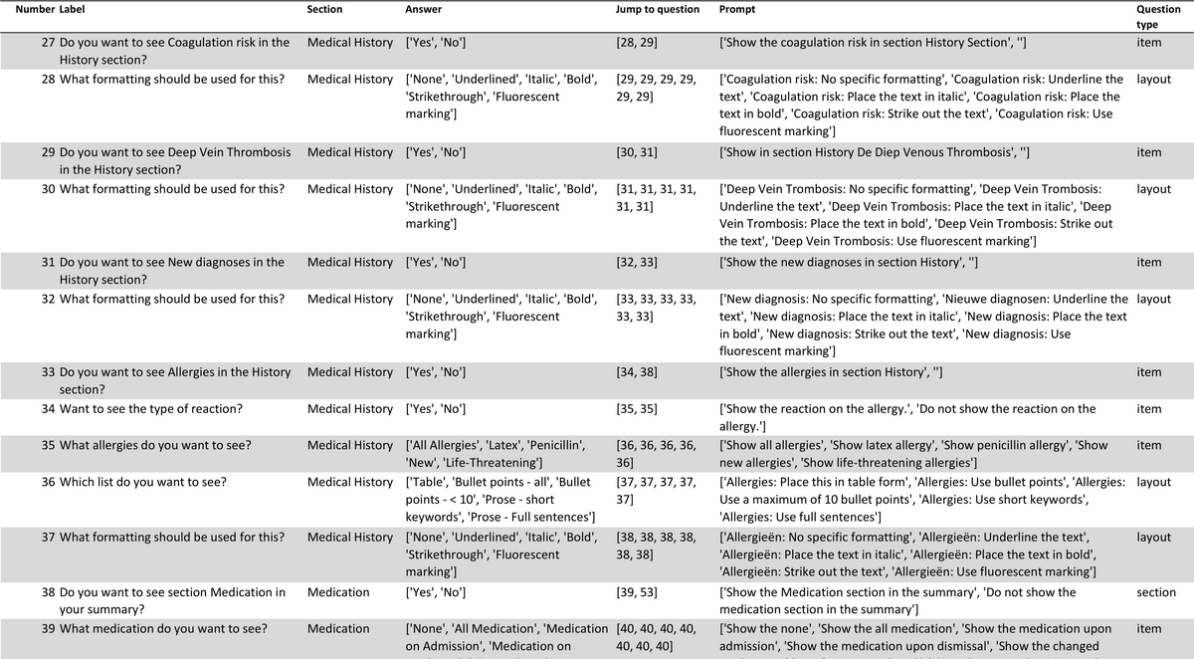
Snippet from the resulting questionnaire list translated into English demonstrating the mapping of provider needs to specific questions and corresponding artificial intelligence prompt template.

After refining the ideas, a structured table was created as the foundation for the questionnaire. These 2 files, along with their interpretations (available only in Dutch), are included in Tables S2 and S3 and Figure S2 in Multimedia Appendix 1. We provide a Python script that can be used to generate a list of questions and the appropriate “jump-to” points where necessary starting from these files. The resulting 110 questions were uploaded to Microsoft Forms, ready to be answered by the participants. The complete list of questions is available in Table S7 in Multimedia Appendix 1, and a (translated) snippet of these questions can be found in Figure 2. In the same script, when creating questions with multiple possible answers, the corresponding prompt for each answer was also added.

Table S8 in Multimedia Appendix 1 shows the number of questions for each section. “Medical history” and “admission history” constituted the largest parts of the questionnaire.

Most health care providers (30/33, 90.9%) were physicians, with over 81.8% (27/33) having more than 5 years of experience (Figure S6 in Multimedia Appendix 1). The “follow-up” section was highly valued by all participants, whereas the “examination” section received the least interest (Figure S7 in Multimedia Appendix 1).

An example of the resulting individualized and final generic prompt can be found in Figure S8 in Multimedia Appendix 1.

**Figure 3.**
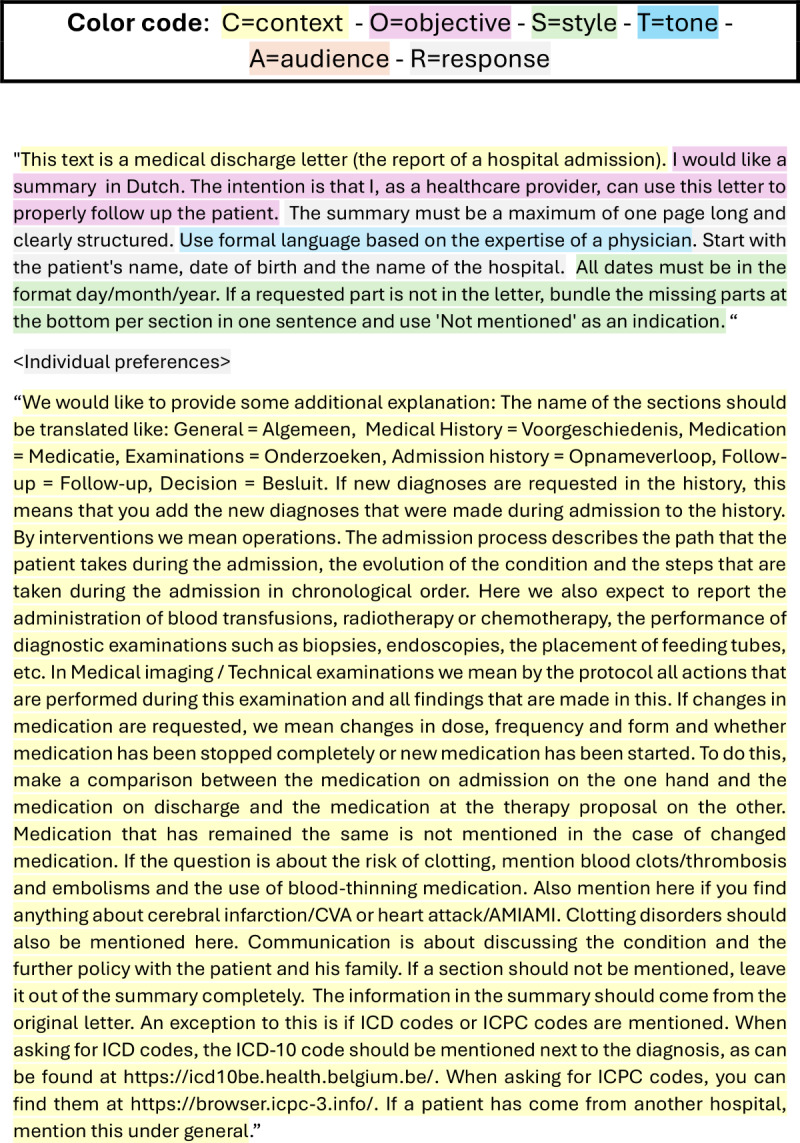
Illustration of the context, objective, style, tone, audience, and response framework application in prompt engineering: integrating health care provider preferences and clinical standards to optimize artificial intelligence–generated discharge summary prompts.

The implementation of the CO-STAR framework produced prompts that effectively reflected both health care provider preferences and established professional standards. The “style” component was defined as “written from the perspective of a medical professional,” and the “tone” element specified the use of formal language. These parameters were selected based on aggregated participant feedback, which underscored the importance of professionalism and clarity in clinical communication. Consequently, the final prompts incorporated user-driven insights alongside best practices, ensuring that AI-generated summaries were both contextually relevant and professionally suitable. A comprehensive overview of the adjustments made to the CO-STAR framework is provided in Figure 3. The prompt structure was enhanced with an introductory segment and a concluding section, whereas the central portion, labeled “<Individual preference>,” reflects findings from the questionnaire.

Table 2 shows the differences in the 10 most common preferences regarding discharge summaries between general practitioners and specialists. General practitioners tend to seek additional details regarding medication and follow-up, whereas specialists more frequently request information pertaining to admission history and examinations.

**Table 2.** Comparison of discharge summary preferences between general practitioners (GPs) and specialists, highlighting the top 20 content areas with the greatest differences in selection rates. Options with negative percentages were favored by specialists, whereas those with positive percentages were preferred by GPs.

Section	Preference	Difference between GPs and specialists (%)
Admission history	Showing acquisition of the interventions performed	−25.3
Admission history	Showing consultations	−14.0
Examinations	Showing the protocol	−13.3
Medical history	Showing life-threatening allergies	−13.3
Examinations	Showing laboratory test results	−12.6
Admission history	Showing main diagnosis	−11.6
Medical history	Showing reaction to allergy	−11.6
Admission history	Showing new diagnoses	−9.8
Examinations	Showing clinical investigation	−8.6
Examinations	Showing medical imaging or technical investigations	−8.6
Follow-up	Showing inclusion in specific care processes	26.8
Follow-up	Showing message for GP	18.7
General	Showing whether the patient is palliative	16.6
Follow-up	Showing the institutions involved	16.6
Medication	Showing changed medication (dose, frequency, and shape)	15.1
Medication	Showing new medication	14.4
Follow-up	Showing in section “follow-up” the communication	13.2
Follow-up	Showing multidisciplinary follow-up (social and home help)	13.2
Medication	Showing stopped medication	12.5
Medication	Showing duration of medication use	12.3

## Discussion

### Principal Findings

From a single workshop with motivated health care providers, it was possible to generate an automated, individualized prompt tailored to their respective needs, enabling the creation of an ideal medical discharge summary. The process began with the identification of key areas that required attention, such as medical history, medication details, and follow-up procedures. Participants provided input on what specific information should be included in each section, ensuring that the final generated summary would be comprehensive and tailored to the needs of health care professionals. To the best of our knowledge, this is the first study to begin with a multidisciplinary workshop involving physicians from diverse specialties and translate their input into tailored AI prompts.

The collaboration between specialists and general practitioners resulted in an exchange of ideas, leading to a broader perspective from both groups. This synergy allowed for a more holistic approach to the creation of the summary, taking into account both the specialized and general aspects of patient information. At the same time, it became clear that reflecting on one’s ideal summary is not always easy—despite it being a personal matter. Sparring with others did not necessarily lead to adopting their viewpoints but proved valuable in helping participants critically reassess and refine their own preferences.

Dividing the scope into layout and content made the resulting ideas more concrete. By separating these 2 elements, participants were able to focus more effectively on each aspect, leading to clearer and more actionable suggestions. This also leads to a clearer prompt for further evaluation possibilities (in future work), providing a solid foundation for future improvements and refinements based on feedback from health care professionals.

Within the workshop, numerous ideas were generated. The open question at the end—“Do you have any additional comments or suggestions regarding this questionnaire? Are there aspects that you think are missing or not sufficiently addressed? Please note them below.”—received responses primarily on practical issues from only a few participants.

During the workshop, it was observed that all groups contributed an equal number of ideas. Most ideas were related to the structure of the generated summary, primarily due to the existence of a single category for the broader theme. The “follow-up” category encompassed the highest number of ideas, although there was significant overlap with other categories. The “communication” category, introduced during the workshop, predominantly contained ideas that pertained to the “follow-up” category. This trend was also evident in the questionnaire, where the “communication” section was the only one indicated as desirable by all participants.

The “medical history” and “admission history” sections often included diverse content, resulting in numerous questions from participants. The “medication” and “follow-up” sections also encompassed a wide range of topics, which necessitated their inclusion as multiple-choice questions. This strategy allowed for a targeted approach, meaning that only 1 detailed question on content was asked for each selected topic (eg, “What information do you want to see for the medication? ‘Start date,’ ‘Duration,’ ‘Dose,’ ‘Time of intake,’ ‘Frequency,’ ‘Form e.g. oral,’ ‘Show drug substance name,’ ‘Clinical effect,’ ‘Criteria for stopping/changing specific medication,’ ‘Reason for starting, stopping, changing’”).

Such a detailed and structured approach ensures that all relevant aspects are thoroughly explored and addressed, providing a comprehensive framework for summarizing medical discharge letters efficiently and effectively.

### Comparison With Prior Work

The most significant refinement aligned with the CO-STAR prompt engineering framework was introduced in the “response” section, with a particular emphasis on clarifying the definitions of key terminology embedded within the prompt. This adjustment is crucial for enhancing the model’s interpretability and ensuring consistent semantic alignment between the prompt and the generated summary. As demonstrated in the work on SARA [13], a generative AI for legal summarization, precise control over terminology and density within prompts directly influences the coherence and legal validity of the summaries produced. Similarly, the Institute of Electrical and Electronics Engineers Computer Society’s International Conference on Computers, Software, and Applications 2024 study [12] on automatic smart contract generation underscores the importance of terminological precision in the response phase to mitigate ambiguity and improve automation reliability. Furthermore, the insights from Teo [14] from the GPT-4 prompt engineering competition highlight how nuanced refinements in prompt structure—especially in the response section—can significantly elevate performance in competitive and real-world applications.

Our findings align with broader trends in health informatics that emphasize the centrality of user needs and adaptive information delivery. For instance, psychoeducational mobile health interventions for parents of small-for-gestational-age fetuses have shown how adaptive, user-centered systems can enhance engagement and outcomes [15]. The ADEPT framework further illustrates how optimizing data flow in dynamic, real-world environments supports effective, personalized care [16]. By translating qualitative input from health care providers into structured, individualized prompts, our framework extends these principles to the domain of clinical AI, ensuring that LLM-generated discharge summaries are not only technically robust but also contextually relevant and responsive to end user needs.

### Limitations

An important aspect of this framework’s broader impact is its adaptability beyond its original Dutch-speaking setting. Future efforts should focus on translation and cultural adjustment of all materials, questionnaires, and prompts used in workshops. This will require close collaboration with language specialists and domain specialists to ensure accurate terminology, workflows, and compliance. Owing to its modular design, the framework allows for easy alignment of categories and prompts with various health care systems’ specific summarization needs. This methodology is applicable across medical specialties by engaging domain experts during assessment and prompt development, ensuring that specialty-specific information needs are addressed. By taking these steps, the framework will become more valuable and help develop AI tools that are effective, equitable, and well-suited to diverse clinical contexts.

Despite aiming for comprehensiveness, the balance between breadth and depth sometimes required trade-offs, prioritizing clarity and efficiency over detailed customization. For instance, preferences were gauged using a multiple-choice question such as “What do you want to see...” followed by only a single question about layout preferences for each item.

This initial prompt is a first step in the improvement in the quality and relevance of AI-generated summaries. It is evident that further refinement is necessary. This ongoing fine-tuning will involve a meticulous evaluation of the generated summaries, taking into account detailed feedback from health care professionals. Such iterative adjustments aim to enhance the precision and contextual accuracy of the summaries, ensuring that they meet the nuanced needs of diverse medical specializations.

### Future Steps

The subsequent phase will involve evaluating the generic and individual prompts, with the assessment being developed collaboratively with the health care providers. The prompt will be broken down into smaller components, generating binary questions regarding layout and more specific inquiries related to content accuracy (eg, if an outcome is incorrect, does it constitute a hallucination?). A collection of representative medical discharge letters will be made available for this purpose. Upon completion of the evaluation, it will likely be necessary to review the prompt to identify opportunities for further optimization and integrate additional content informed by the evaluation findings.

### Conclusions

This study presents a novel, systematic framework for developing individualized prompts to summarize medical discharge letters grounded in the real-world needs of health care providers. A key strength of this approach lies in the deliberate separation between layout and content preferences, which proved highly effective in capturing both structural and informational expectations.

By translating qualitative input into structured, personalized prompts, this framework aims to improve the relevance and usability of AI-generated summaries and serve as a replicable model for diverse clinical and research settings, although empirical validation remains necessary. The framework supports applications in data collection, data analysis, and personalized feedback generation. This study is limited to the design and implementation of a human-centered prompt construction pipeline in a specific multicenter setting; direct evaluation of AI-generated outputs and broader scalability will be addressed in future work.

## Supplementary material

10.2196/80613 Multimedia Appendix 1 Overview of all supplementary materials, including workshop energizer results, input questionnaire structure, informed consent template, participant characteristics, word‑cloud analysis, padlet examples, workshop output (in Dutch), full questionnaire, and questionnaire results.

10.2196/80613 Checklist 1 STROBE checklist.

## Abbreviations

AI: artificial intelligence
CO-STAR: context, objective, style, tone, audience, and response
FRAIT: Framework & Implementation of AI Tools
LLM: large language model
STROBE: Strengthening the Reporting of Observational Studies in Epidemiology
