# Supplementary material for: Tailoring Discharge Summaries to Health Care Providers’ Needs (Part 1 of the Framework and Implementation of AI Tools Project): User-Centered Design Approach
Source: JMIR Med Inform. 2026 Mar 4;14:e80613. doi: 10.2196/80613 (PMC12978883; doi:10.2196/80613)
Supplement: Multimedia Appendix 1 [file medinform-v14-e80613-s001.pdf]

Multimedia Appendix 1

Overview

Appendix A – Workshop Energizer 2

A.1 Table S1 - Results energizer 2

A.2 Figure S1 - Example Energizer 3

Appendix B – Input questionnaire 4

B.1 Table S2 Input questionnaire - basic 4

B.2 Table S3 Input questionnaire – details 6

B.3 Figure S2 Interpretation Input B.1 (Table S2) & B.2 (Table S3) 10

Appendix C – Informed consent 11

C.1 Figure S3 Informed Consent 11

Appendix D – Participants and organization 12

D.1 Table S4 Participants by Institutions 12

D.2 Table S5 Participants by organization, role, years of experience, workshop attendance and filled in questionnaire 13

Appendix E – Word cloud results and ideas 14

E.1 Figure S4 - Word cloud results and ideas : Visualization of key themes and concepts identified by health care providers during the Framework and Implementation of AI Tools workshop, highlighting the most frequently mentioned elements for discharge summary design. 14

Appendix F – example padlet 15

F.1 Figure S5 Example padlet 15

Appendix G – Workshop output – all resulting ideas (Dutch) 16

G.1 Table S6 Workshop output – all resulting ideas (Dutch) 16

Appendix H – Questionnaire 24

H.1 Table S7 Overview questionnaire (Dutch) 24

H.2 Table S8 Questionnaire - number of questions by each section 32

Appendix I – Results questionnaire 33

I.1 Figure S6 Type of Health Care Provider and Years of Experience 33

I.2 Figure S7 Percentage affirmative responses to whether participants wanted specific sections included in the summary 33

Appendix J – example individual prompt (English) 34

J.1 Figure S8 Example individual prompt (English) 34

Appendix A – Workshop Energizer

A.1 Table S1 - Results energizer

Nr.	Question	Description	Blue	Yellow	Green
**1**	Type health care provider?	Who is present today?	General Practitioner	Specialist	Other
			8 (33%)	14 (58%)	2 (8%)
**2**	During the work context, how many medical letters do you read?	Progress notes, discharge letters? Frequency of need to view, review medical texts	A lot (> 15)	Average (5-15)	Little (<5)
			20 (83%)	2 (8%)	2 (8%)
**3**	During the work context: how do you experience the accessibility of those medical letters	Process of reviewing medical texts do I experience as?	At a glance	workable but...	I waste a lot of unnecessary time
			0 (0%)	15 (63%)	9 (38%)
**4**	Who uses LLMs	Who uses LLMs like ChatGPT, CoPilot?	Never	Private	Private & Clinical
			4 (17%)	10 (42%)	10 (42%)
**5**	LLM output step 1	This summary is?	Excellent	Workable but...	Not usable
			2 (8%)	22 (92%)	0 (0%)
**6**	LLM output step 2	This summary is?	Excellent	Workable but...	Not usable
			2 (8%)	22 (92%)	0 (0%)
**7**	LLM output step 3	This summary is?	Excellent	Workable but...	Not usable
			0 (0%)	18 (75%)	6 (25%)

Table S1 Results from the energizer questions: who is present in this workshop and what is the experience level with LLMs.

A.2 Figure S1 - Example Energizer

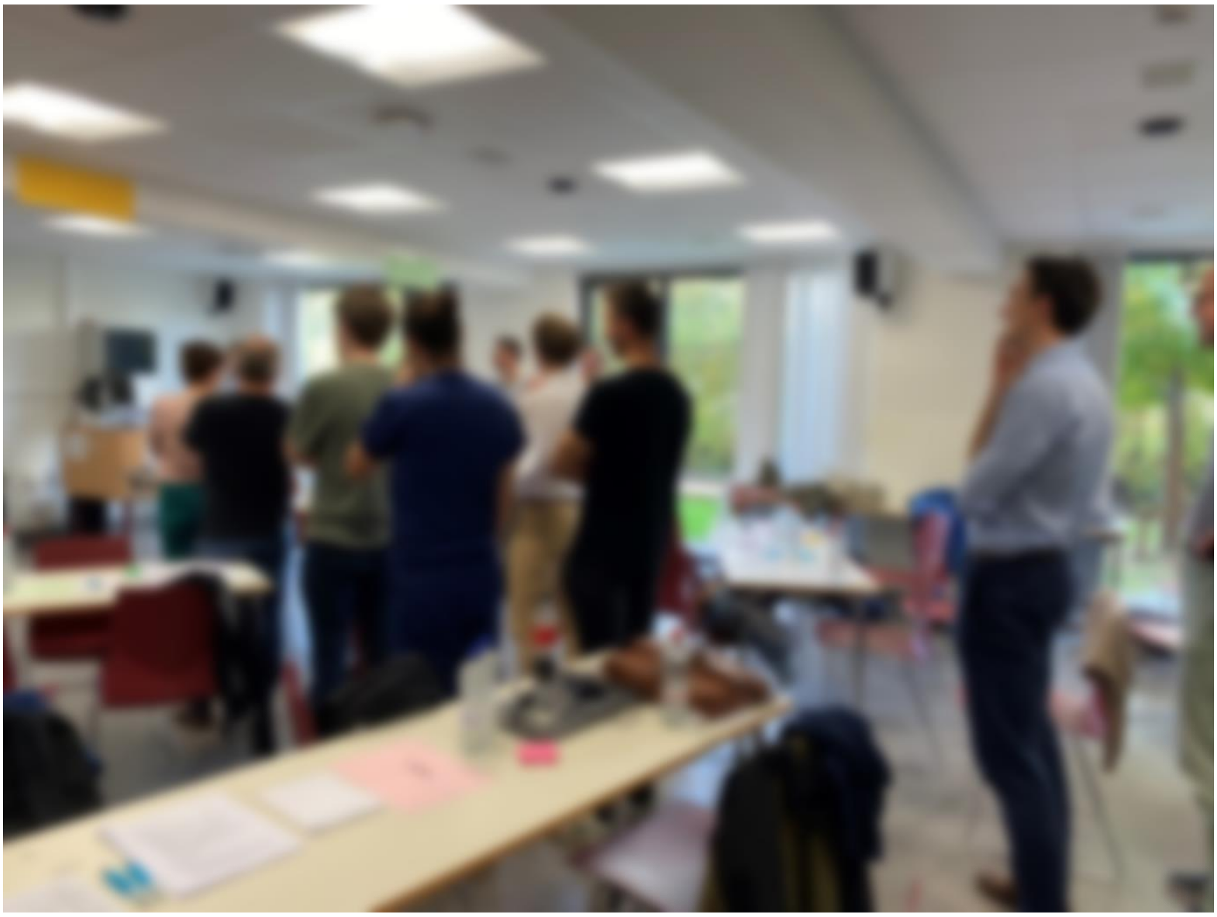

Appendix B – Input questionnaire

B.1 Table S2 Input questionnaire - basic

Sectie	Basisvraag	Niveau3	Niveau4	Niveau5	Niveau6	Niveau7
Algemeen	##ALGEMEEN##_Onderdeel	##ALGEMEEN##_Opmaak	##OPMAAK##	##LIJST##		
Voorgeschiedenis	##SpecifiekeLIJST##	##LIJST##				
Voorgeschiedenis	een Specifiek deel van de Voorgeschiedenis apart opgelijst	##Voorgeschiedenis##	##OPMAAK##			
Voorgeschiedenis	ICD codes					
Voorgeschiedenis	ICPC codes					
Voorgeschiedenis	Gebruik van tabak	##OPMAAK##				
Voorgeschiedenis	Gebruik van alcohol	##OPMAAK##				
Voorgeschiedenis	BMI	##OPMAAK##				
Voorgeschiedenis	Geteste immuniteiten	##LIJST##	##OPMAAK##			
Voorgeschiedenis	Stollingsrisico	##OPMAAK##				
Voorgeschiedenis	Diep Veneuze Trombose	##OPMAAK##				
Voorgeschiedenis	Nieuwe diagnosen	##OPMAAK##				
Voorgeschiedenis	Allergieën	##SoortReactie##	##Allergie##	##LIJST##	##OPMAAK##	
Medicatie	##WelkeMedicatie##_Onderdeel	##WelkeMedicatie##_Opmaak	##OPMAAK##	##GegevensMedicatie##	##LIJST##	##TIJD##
Medicatie	##SpecifiekeMedicatie##	##WAAR##	##OPMAAK##			
Medicatie	Alternatieve medicatie (bijv. indien bijwerkingen optreden)	##OPMAAK##				
Medicatie	Bijwerkingen / interacties	##OPMAAK##				
Medicatie	Kleurcodes					
Onderzoeken	Klinisch onderzoek	##LIJST##	##TIJD##	##DETAILS MODUS##		
Onderzoeken	Laboratoriumresultaten	##LIJST##	##TIJD##	##DETAILS MODUS##		
Onderzoeken	Medische beeldvorming / Technische onderzoeken	##PROTOCOL##	##DETAILS MODUS##			
Onderzoeken	Pre-opname onderzoeken (bijv. ECG)	##LIJST##				
Opnameverloop	Hoofddiagnose	##OPMAAK##				

Opnameverloop	Nieuwe diagnosen	##SpecifiekeLIJST##	##LIJST##	##OPMAAK##		
Opnameverloop	ICD codes					
Opnameverloop	Transfers tussen afdelingen	##LIJST##	##OPMAAK##			
Opnameverloop	Consultaties	##LIJST##	##TIJD##	##OPMAAK##		
Opnameverloop	Uitgevoerde ingrepen	##SpecifiekeOperaties##	##OPMAAK##			
Opnameverloop	Uitgevoerde ingrepen	Verslagen	##LIJST##	##TIJD##	##DETAILS MODUS##	
Opnameverloop	Uitgevoerde ingrepen	Vreemd materiaal	##LIJST##	##TIJD##	##DETAILS MODUS##	
Follow-up	##FOLLOWUP##_Onderdeel	##FOLLOWUP##_Opmaak	##OPMAAK##	##LIJST##	##TIJD##	
Follow-up	Communicatie	##Communicatie##	##OPMAAK##			
Follow-up	Psychosociale context	##LIJST##	##OPMAAK##			
Besluit	enkel nieuwe items (diagnosen, medicatie, …)	##LIJST##				
Besluit	##SECTIES##	##LIJST##				

B.2 Table S3 Input questionnaire – details

Tag	Optie	Prompt_Nl	Prompt_Eng
##WAAR##	Onderaan deze sectie	Plaats dit onderaan deze sectie	Put this on the bottom of this section
##WAAR##	Bovenaan deze sectie	Plaats dit bovenaan deze sectie.	Place this at the top of this section
##WAAR##	Geen voorkeur	Gebruik een nieuwe alinea.	Use a new paragraph.
##OPMAAK##	Geen	Geen specifieke opmaak	No specific formatting
##OPMAAK##	Onderlijnd	Onderlijn de tekst	Underline the text
##OPMAAK##	Italic	Onderlijn de tekst	Place the text in italic
##OPMAAK##	Vet	Plaats de tekst in het vet	Place the text in bold
##OPMAAK##	Doorgehaald	Haal de tekst door	Strike out the text
##OPMAAK##	Fluo markering	Gebruik fluo markering	Use fluorescent marking
##LIJST##	Tabel	Plaats dit in tabelvorm	Place this in table form
##LIJST##	Bulletpoints - alle	Gebruik bulletpoints	Use bullet points
##LIJST##	Bulletpoints - < 10	Gebruik maximum 10 bulletpoints	Use a maximum of 10 bullet points
##LIJST##	Proza - korte kernwoorden	Gebruik korte kernwoorden	Use short keywords
##LIJST##	Proza - Volzinnen	Gebruik volzinnen	Use full sentences
##TIJD##	Geen specifieke	Geen specifieke ordening	No specific ordening
##TIJD##	Alfabetisch	Zet de items alfabetisch	Arrange the items alfabetic
##TIJD##	Chronologisch	Zet de items chronologisch	Arrange the items chronologically
##TIJD##	Per orgaansysteem	Zet de items thematisch	Arrange the items thematically
##DETAILS MODUS##	Geen	Toon geen details	Do not show details
##DETAILS MODUS##	(Hyper)links	Gebruik (hyper)links om de details weer te geven.	Use (hyper)links to view the details.
##DETAILS MODUS##	Tabbladen	Gebruik tabbladen om de details weer te geven.	Use tabs to view the details.
##DETAILS MODUS##	In tekst zelf	Zet de details in de tekst zelf.	Put the details in the text itself.
##SECTIES##	Algemeen (bijv. ligduur, opnamedatum, ...)	Toon de sectie algemeen	Show the section General
##SECTIES##	Voorgeschiedenis	Toon de sectie voorgeschiedenis	Show the section Medical History
##SECTIES##	Medicatie	Toon de sectie medicatie	Show the section Medication

##SECTIES##	Onderzoeken	Toon de sectie onderzoeken	Show the section Examinations
##SECTIES##	Opnameverloop	Toon de sectie opnameverloop	Show the admission history
##SECTIES##	Follow-up	Toon de sectie follow-up	Show the section Follow-up
##PROTOCOL##	Ja	Toon het protocol.	Show the protocol
##PROTOCOL##	Nee	Toon het protocol niet	Do not show the protocol
##SoortReactie##	Ja	Toon het soort reactie op de allergie.	Show the reaction on the allergy.
##SoortReactie##	Nee	Toon het soort reactie niet op de allergie	Do not show the reaction on the allergy.
##ALGEMEEN##	Geen		
##ALGEMEEN##	Ligduur	Toon de ligduur	Show the length of stay
##ALGEMEEN##	Opnamedatum	Toon de opnamedatum	Show the admission date
##ALGEMEEN##	Ontslagdatum	Toon de ontslagdatum	Show the discharge date
##ALGEMEEN##	Reden van opname	Toon de reden van opname	Show the reason of admission
##ALGEMEEN##	Type brief (ontslagbrief, consultatiebrief)	Toon de type brief (ontslagbrief, consultatiebrief)	Show the type of letter (discharge letter, consultation letter)
##ALGEMEEN##	Nieuwe patiënt	Toon dat dit een nieuwe patient	Show if this is a new patient
##ALGEMEEN##	Discipline	Toon de discipline	Show the specialism
##ALGEMEEN##	Naam en gegevens behandelende arts	Toon de naam en gegevens behandelende arts	Show the name and details of the attending physician
##ALGEMEEN##	Opnamedienst	Toon de opnamedienst	Show the admission ward
##ALGEMEEN##	Bestemming van de patiënt na ontslag	Toon de bestemming van de patiënt	Show the destination of the patient
##ALGEMEEN##	Palliatief	Toon of de patient palliatief is	Show if patient is palliative
##ALGEMEEN##	DNR	Toon de DNR code	Show the DNR code
##ALGEMEEN##	Overleden (aanduiden met kruisje naast de naam)	Toon of de patient overleden is (aanduiden met kruisje naast de naam)	Show is the patient has died (show with a cross mark next to the patients name)
##Voorgeschiedenis##	Chirurgische Voorgeschiedenis	Toon de chirurgische voorgeschiedenis	Show the surgical medical history
##Voorgeschiedenis##	Obstetrische Voorgeschiedenis (GPA, Laatste terugbetaalde PAP, Termijn partus, Zwangerschapcomplicaties, Modus partus)	Toon de obstetrische voorgeschiedenis (gpa, laatste terugbetaalde pap, termijn partus, zwangerschapcomplicaties, modus partus)	Show obstetric history (GPA, last reimbursed pap, term of parturition, pregnancy complications, mode of parturition)
##Voorgeschiedenis##	Niet-chirurgische Voorgeschiedenis (o.a. cardiologisch, oncologisch, …)	Toon de niet-chirugische voorgeschiedenis (o.a. cardiologisch, …)	Show non-surgical history (including cardiology, …)
##Allergie##	Alle allergieën	Toon alle allergieën	Show all allergies
##Allergie##	Latex	Toon de latex allergie	Show latex allergy
##Allergie##	Penicilline	Toon de penicilline allergie	Show penicillin allergy

##Allergie##	Nieuwe	Toon de nieuwe allergieën	Show new allergies
##Allergie##	Levensbedreigende	Toon de levensbedreigende allergieën	Show life-threatening allergies
##WelkeMedicatie##	Geen		
##WelkeMedicatie##	Alle Medicatie	Toon alle medicatie	Show all medication
##WelkeMedicatie##	Medicatie bij opname	Toon de medicatie bij opname	Show medication upon admission
##WelkeMedicatie##	Medicatie bij ontslag	Toon de medicatie bij ontslag	Show medication upon discharge
##WelkeMedicatie##	Gewijzigde Medicatie (dosis, frequentie, vorm)	Toon de gewijzigde medicatie	Show changed medication
##WelkeMedicatie##	Gestopte Medicatie	Toon de gestopte medicatie	Show stopped medication
##WelkeMedicatie##	Gestarte / nieuwe medicatie	Toon de gestarte / nieuwe medicatie	Show started/new medication
##SpecifiekeMedicatie##	Geen		
##SpecifiekeMedicatie##	Medicatie beperkt in tijdsduur	Highlight de tijdelijke medicatie	Highlight temporary medication
##SpecifiekeMedicatie##	Chronische medicatie	Highlight de chronische medicatie	Highlight chronic medication
##SpecifiekeMedicatie##	Bloedverdunners	Highlight de bloedverdunners	Highlight blood thinners
##SpecifiekeMedicatie##	Chirurgische medicatie	Highlight de chirurgische medicatie (bisfosfonaten, ontstollende)	Highlight surgical medication (bisphosphonates, anticoagulants)
##SpecifiekeMedicatie##	Psychofarmaca	Highlight de psychofarmaca	Highlight psychotropic drugs
##GegevensMedicatie##	Startdatum	Toon de startdatum	Show start date
##GegevensMedicatie##	Duur	Toon de duur van de medicatie	Show duration of medication
##GegevensMedicatie##	Dosis	Toon de dosis van de medicatie	Show dosage of medication
##GegevensMedicatie##	Tijdstip van inname	Toon het tijdstip van inname van de medicatie	Show the time of intake of the medication
##GegevensMedicatie##	Frequentie	Toon de frequentie van de medicatie	Show the frequency of the medication
##GegevensMedicatie##	Vorm (oraal, …)	Toon de vorm (bijv. oraal) van de medicatie	Show the form (e.g. oral) of the medication
##GegevensMedicatie##	Stofnaam medicatie weergeven	Geef de stofnaam van de medicatie	Show substance name of medication
##GegevensMedicatie##	Klinisch effect	Toon het klinisch effect van de medicatie	Show clinical effect of medication
##GegevensMedicatie##	Criteria om specifieke medicatie te stoppen / wijzigen	Toon de criteria om de medicatie te stoppen of the wijzigen.	Show the criteria to stop or adjust the medication
##GegevensMedicatie##	Reden van start, stop, wijziging	Toon de reden om medicatie te starten, stoppen of wijzigen.	Show the reason to start, stop or adjust the medication
##Communicatie##	De alarmsignalen voor de patiënt	Toon de de alarmsignalen voor de patient	Show alarm signals for patient
##Communicatie##	Boodschap voor Huisarts	Toon de boodschap voor huisarts	Show message for GP
##Communicatie##	Diagnose besproken met patiënt	Toon de diagnose besproken met patient	Show diagnosis discussed with patient
##Communicatie##	Aanduiding Informed Consent gegeven	Toon de aanduiding informed consent gegeven	Show indication informed consent given

##SpecifiekeLIJST##	Chronologisch	Toon dit chronologisch	Show chronologically
##SpecifiekeLIJST##	Per orgaanproblematiek	Toon dit per orgaanproblematiek	Show per organ problem
##SpecifiekeLIJST##	Per specialisme	Toon dit per specialisme	Show per specialty
##SpecifiekeOperaties##	Geen		
##SpecifiekeOperaties##	Urologische-Gynaecologische operaties	Highlight de urologische-gynaecologische operaties	Highlight de urological-gynecological operations
##SpecifiekeOperaties##	Gastrointestinale operaties	Highlight de gastrointestinale operaties	Highlight gastrointestinal operations
##SpecifiekeOperaties##	Hart- en vaatoperaties	Highlight de hart- en vaat operaties	Highlight cardiovascular operations
##SpecifiekeOperaties##	Oncologische operaties	Highlight de oncologische operaties	Highlight oncological operations
##SpecifiekeOperaties##	Neurologische operaties	Highlight de neurologische operaties	Highlight neurological operations
##FOLLOWUP##	Geen		
##FOLLOWUP##	Geplande onderzoeken	Toon de geplande onderzoeken	Show the planned examinations
##FOLLOWUP##	Geplande consultaties	Toon de geplande consultaties	Show the planned consultations
##FOLLOWUP##	Opvolgafspraken	Toon de opvolg afspraken	Show the follow-up appointments
##FOLLOWUP##	TODO lijst (wie-wat-wanneer/ aandachtspunten per zorgverlener)	Toon de TODO-lijst (wie-wat-wanneer/ aandachtspunten per todo lijst wie wat wanneer aandachtspunten per zorgverlener	Show the TODO list (who-what-when/ points of attention per todo list who what when points of attention per care provider
##FOLLOWUP##	Multidisciplinaire opvolging (sociaal, thuishulp)	Toon multidisciplinaire opvolging sociaal thuishulp	Show multidisciplinary follow-up social home care
##FOLLOWUP##	Betrokken instellingen	Toon de betrokken instellingen	Show the institutions involved
##FOLLOWUP##	Inclusie in specifieke zorgtrajecten	Toon de inclusie in specifieke zorgtrajecten	Show the inclusion in specific care pathways

B.3 Figure S2 Interpretation Input B.1 (Table S2) & B.2 (Table S3)

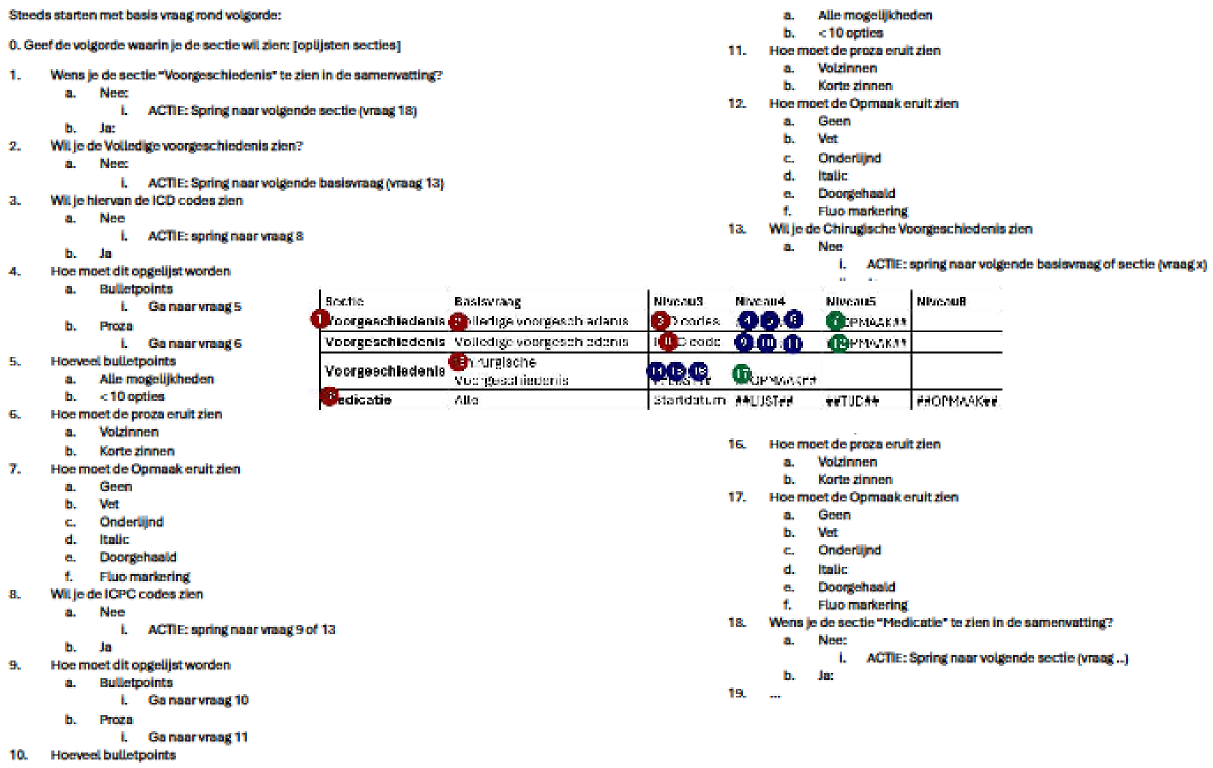

Steeds starten met basis vraag rond volgorde:

0. Geef de volgorde waarin je de sectie wil zien: [oplijsten secties]

1. Wens je de sectie "Voorgeschiedenis" te zien in de samenvatting?
   a. Nee:
      i. ACTIE: Spring naar volgende sectie (vraag 18)
   b. Ja:
2. Wil je de Volledige voorgeschiedenis zien?
   a. Nee:
      i. ACTIE: Spring naar volgende basisvraag (vraag 13)
3. Wil je hiervan de ICD codes zien
   a. Nee
      i. ACTIE: spring naar vraag 8
   b. Ja
4. Hoe moet dit opgelijst worden
   a. Bulletpoints
      i. Ga naar vraag 5
   b. Proza
      i. Ga naar vraag 6
5. Hoeveel bulletpoints
   a. Alle mogelijkheden
   b. < 10 opties
6. Hoe moet de proza eruit zien
   a. Volzinnen
   b. Korte zinnen
7. Hoe moet de Opmaak eruit zien
   a. Geen
   b. Vet
   c. Onderlijnd
   d. Italic
   e. Doorgehaald
   f. Fluo markering
8. Wil je de ICPC codes zien
   a. Nee
      i. ACTIE: spring naar vraag 9 of 13
   b. Ja
9. Hoe moet dit opgelijst worden
   a. Bulletpoints
      i. Ga naar vraag 10
   b. Proza
      i. Ga naar vraag 11
10. Hoeveel bulletpoints
    a. Alle mogelijkheden
    b. < 10 opties
11. Hoe moet de proza eruit zien
    a. Volzinnen
    b. Korte zinnen
12. Hoe moet de Opmaak eruit zien
    a. Geen
    b. Vet
    c. Onderlijnd
    d. Italic
    e. Doorgehaald
    f. Fluo markering
13. Wil je de Chirugische Voorgeschiedenis zien
    a. Nee
       i. ACTIE: spring naar volgende basisvraag of sectie (vraag x)

Sectie	Basisvraag	Niveau3	Niveau4	Niveau5	Niveau6
1 [illegible]oorgeschiedenis	2 [illegible]olledige voorgeschiedenis	3 [illegible] codes	4 5 6 [illegible]	7 [illegible]OPMAAK##	
Voorgeschiedenis	Volledige voorgeschiedenis	8 [illegible] code	9 10 11 [illegible]	12 [illegible]OPMAAK##	
Voorgeschiedenis	13 [illegible]rurgische Voorgeschiedenis	14 15 16 [illegible]	17 [illegible]OPMAAK##		
[illegible]edicatie	Alle	Startdatum	##LIJST##	##TIJD##	##OPMAAK##

16. Hoe moet de proza eruit zien
    a. Volzinnen
    b. Korte zinnen
17. Hoe moet de Opmaak eruit zien
    a. Geen
    b. Vet
    c. Onderlijnd
    d. Italic
    e. Doorgehaald
    f. Fluo markering
18. Wens je de sectie "Medicatie" te zien in de samenvatting?
    a. Nee:
       i. ACTIE: Spring naar volgende sectie (vraag ...)
    b. Ja:
19. ...

Appendix C – Informed consent

C.1 Figure S3 Informed Consent

Informatiebrief voor deelnemers aan Project 'Framework & Implementatie AI Tools' (FRAIT)

Beste,

U wordt uitgenodigd om deel te nemen aan een klinische studie. Neem, voor u beslist om deel te nemen aan deze studie, voldoende tijd om deze informatiebrief aandachtig te lezen en dit te bespreken met de projectcoördinator, of met andere personen binnen het project. Neem ook de tijd om vragen te stellen indien er onduidelijkheden zijn of indien u bijkomende informatie wenst. Dit proces wordt "informed consent" of "geïnformeerde toestemming" voor deelname aan het project genoemd. Eens u beslist heeft om deel te nemen aan de studie zal men u vragen om het toestemmingsformulier op de laatste pagina te ondertekenen.

We starten alvast met enkele termen uit te leggen:

- **Large Language Model** = Groot taalmodel**:** De 'brein' van de computer, die taal begrijpt en kan genereren.
- **Prompt:** De 'vraag' die je aan het brein stelt.
- **Geavanceerde prompt engineering tool:** Het 'gereedschap' om de vraag zo precies mogelijk te stellen

WAT IS HET DOEL VAN DE STUDIE?

Het primaire doel van dit project is het maximaal benutten van een reeds bestaande Large Language Model (LLM) tool binnen de medische sector. Dit wordt verwezenlijkt door de ontwikkeling van een geavanceerde prompt engineering tool, die - rekening houdend met de unieke behoeften van elke zorgverlener - op maat gemaakte en accurate prompts genereert. Deze tool zal voor elke specifieke situatie of vraag van diverse zorgprofessionals de meest geschikte en effectieve prompt samenstellen. De nadruk ligt hierbij niet enkel op de technische integratie van de tool, maar ook op de operationele, ethische en praktische facetten van het toepassen van dit geavanceerde systeem binnen de dynamische en complexe context van een ziekenhuisomgeving.

Een cruciaal onderdeel van dit proces is het ontwikkelen van een validatiesysteem voor continue monitoring en evaluatie van de prompts en de daarbij horende output van de LLM-tool. Dit systeem stelt ons in staat om de betrouwbaarheid en veiligheid van het gebruik van de LLM-tool te waarborgen.

Deze integrale benadering streeft naar een doeltreffende en ethisch verantwoorde uitrol van de LLM-tool, met als doel een handleiding te ontwikkelen. Deze handleiding dient om andere ziekenhuizen en zorginstellingen te begeleiden en hen van cruciale best practices en geleerde lessen te voorzien, zodat zij vergelijkbare tools succesvol kunnen toepassen. De verspreiding van deze kennis zal plaatsvinden via workshops aan het einde van het project. Hierdoor levert het project een essentiële bijdrage aan de verbetering van de patiëntenzorg en de ondersteuning van zorgprofessionals.

De opdrachtgever van deze studie is UZ Gent.

Appendix D – Participants and organization

D.1 Table S4 Participants by Institutions

Organization	n	%
AZ Oudenaarde	6	23%
AZ Sint-Lucas Gent	5	19%
Huisartsenvereniging Gent *	7	27%
Huisartsenkring Schelde Leie *	2	8%
Ghent University Hospital	6	23%
**Total**	**26**	**100%**

Legend: * = GP organization

D.2 Table S5 Participants by organization, role, years of experience, workshop attendance and filled in questionnaire

Organization	Type	Experience	Workshop	Questionnaire	n
AZ Oudenaarde	Specialist	2-5	Yes	Yes	1
	Specialist	5-10	Yes	Yes	1
	Specialist	>10	Yes	Yes	4
AZ Sint-Lucas Gent	Other healthcare provider	>10	No	No	1
	Specialist	2-5	Yes	Yes	3
	Specialist	5-10	No	Yes	2
	Specialist	>10	Yes	Yes	1
Ghent University Hospital	GP	>10	Yes	Yes	1
	Other healthcare provider	>10	No	Yes	1
	Specialist	5-10	No	Yes	1
	Specialist	5-10	Yes	Yes	1
	Specialist	>10	Yes	Yes	4
Huisartsenvereniging Gent	GP	2-5	Yes	Yes	1
	GP	5-10	No	Yes	2
	GP	5-10	Yes	Yes	3
	GP	>10	No	Yes	2
	GP	>10	Yes	Yes	2
	Other healthcare provider	2-5	Yes	Yes	1
Huisartsenkring Schelde Leie	GP	5-10	Yes	Yes	2

Legend: GP = General Practitioner

Appendix E – Word cloud results and ideas

E.1 Figure S4 - Word cloud results and ideas : Visualization of key themes and concepts identified by health care providers during the Framework and Implementation of AI Tools workshop, highlighting the most frequently mentioned elements for discharge summary design.

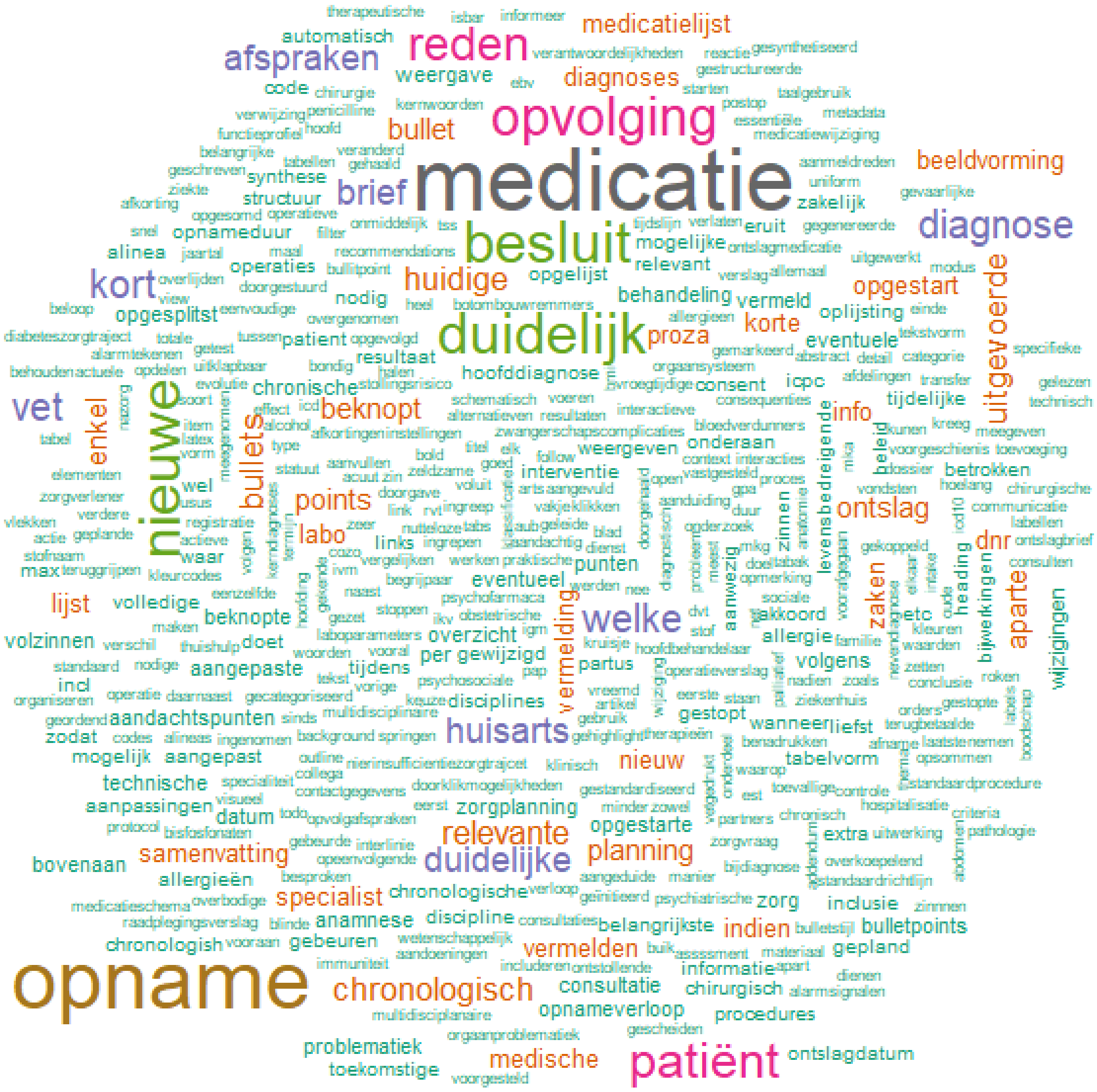

Appendix F – example padlet

F.1 Figure S5 Example padlet

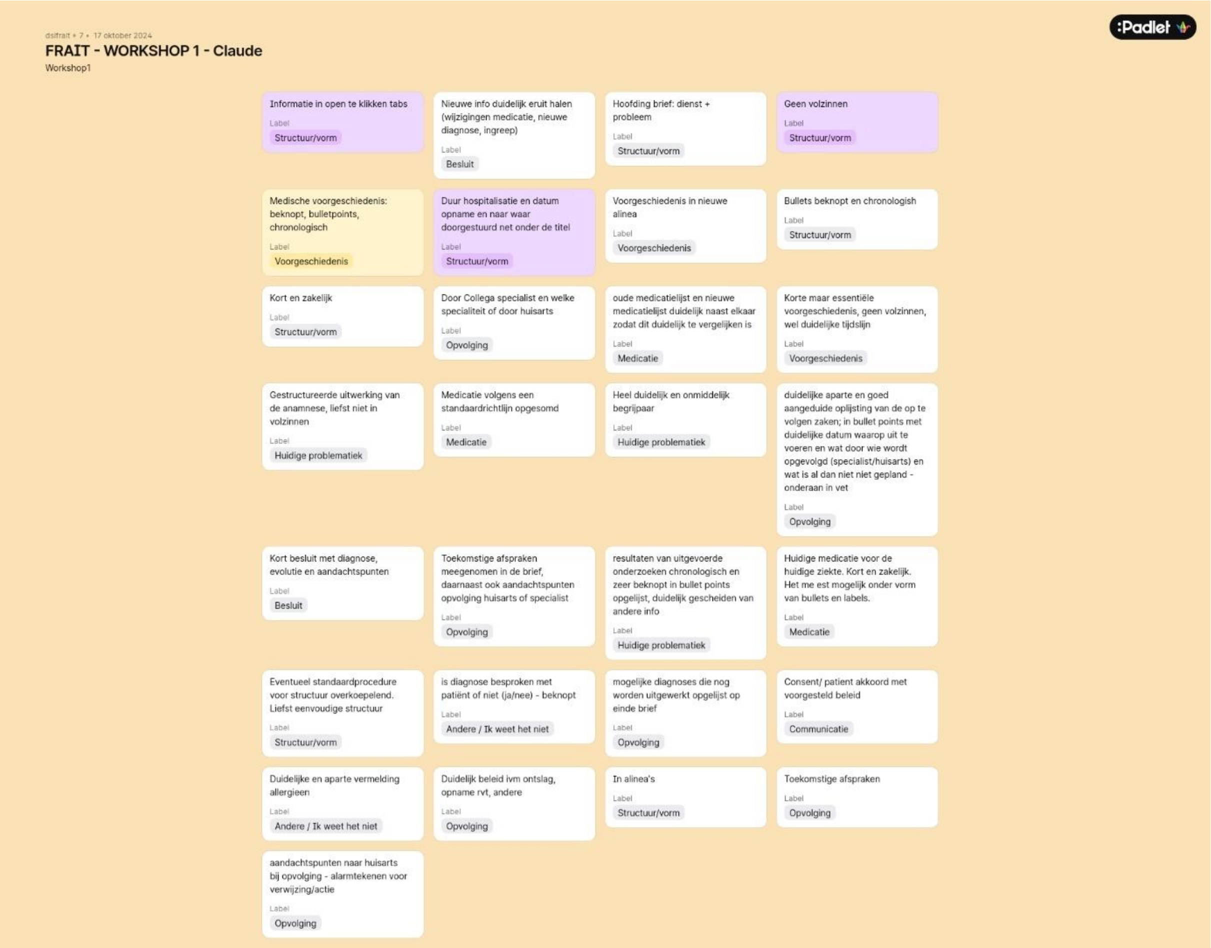

Appendix G – Workshop output – all resulting ideas (Dutch)

G.1 Table S6 Workshop output – all resulting ideas (Dutch)

Id	Number by group	text	label	group
1	1	Toevoeging van ICD ICPC codes	Structuur/vorm	group 1
2	2	Volledige voorgeschiedenis maar wel synthese	Voorgeschiedenis	group 1
3	3	Actuele medicatie lijst bij verlaten ziekenhuis	Medicatie	group 1
4	4	Het moet duidelijk zijn wat nieuw is opgestart, vastgesteld, gewijzigd	Structuur/vorm	group 1
5	5	Chronologische voorgeschiedenis	Voorgeschiedenis	group 1
6	6	Postop orders weergeven	Besluit	group 1
7	7	Nieuwe diagnose in vakje voorgeschiedenis zetten zodat dit in nieuw dossier kan overgenomen worden	Voorgeschiedenis	group 1
8	8	Relevante technische onderzoeken, niet allemaal	Huidige problematiek	group 1
9	9	Opname reden	Huidige problematiek	group 1
10	10	Reden van opname gesynthetiseerd	Huidige problematiek	group 1
11	11	Geen oplijsting van opeenvolgende consultaties aub	Structuur/vorm	group 1
12	12	Geen addendum aan raadplegingsverslag	Besluit	group 1
13	13	Bullets	Huidige problematiek	group 1
14	14	Nieuwe zaken in het vet	Structuur/vorm	group 1
15	15	Bullitpoint waar overzicht nodig is	Besluit	group 1
16	16	Doorklikmogelijkheden naar onderzoeken, beeldvorming, labo	Huidige problematiek	group 1
17	17	Reden van opname moet er uit springen	Structuur/vorm	group 1
18	18	Opnameverloop vooral de belangrijke zaken	Huidige problematiek	group 1
19	19	Nieuwe allergieën benadrukken	Opvolging	group 1
20	20	Nieuwe chronische aandoeningen	Opvolging	group 1

21	21	Usus tabak en alcohol	Voorgeschiedenis	group 1
22	22	Duidelijke communicatie van doorgave van zorg	Opvolging	group 1
23	23	Welke instellingen betrokken bij opvolging	Opvolging	group 1
24	24	Opvolgafspraken	Opvolging	group 1
25	25	Multidisciplinaire nazorg	Opvolging	group 1
26	1	Informatie in open te klikken tabs	Structuur/vorm	group 2
27	2	Nieuwe info duidelijk eruit halen (wijzigingen medicatie, nieuwe diagnose, ingreep)	Besluit	group 2
28	3	Hoofding brief: dienst + probleem	Structuur/vorm	group 2
29	4	Geen volzinnen	Structuur/vorm	group 2
30	5	Medische voorgeschiedenis: beknopt, bulletpoints, chronologisch	Voorgeschiedenis	group 2
31	6	Duur hospitalisatie en datum opname en naar waar doorgestuurd net onder de titel	Structuur/vorm	group 2
32	7	Voorgeschiedenis in nieuwe alinea	Voorgeschiedenis	group 2
33	8	Bullets beknopt en chronologish	Structuur/vorm	group 2
34	9	Kort en zakelijk	Structuur/vorm	group 2
35	10	Door Collega specialist en welke specialiteit of door huisarts	Opvolging	group 2
36	11	oude medicatielijst en nieuwe medicatielijst duidelijk naast elkaar zodat dit duidelijk te vergelijken is	Medicatie	group 2
37	12	Korte maar essentiële voorgeschiedenis, geen volzinnen, wel duidelijke tijdslijn	Voorgeschiedenis	group 2
38	13	Gestructureerde uitwerking van de anamnese, liefst niet in volzinnen	Huidige problematiek	group 2
39	14	Medicatie volgens een standaardrichtlijn opgesomd	Medicatie	group 2
40	15	Heel duidelijk en onmiddelijk begrijpaar	Huidige problematiek	group 2
41	16	duidelijke aparte en goed aangeduide oplijsting van de op te volgen zaken; in bullet points met duidelijke datum waarop uit te voeren en wat door wie wordt opgevolgd (specialist/huisarts) en wat is al dan niet niet gepland - onderaan in vet	Opvolging	group 2
42	17	Kort besluit met diagnose, evolutie en aandachtspunten	Besluit	group 2
43	18	Toekomstige afspraken meegenomen in de brief, daarnaast ook aandachtspunten opvolging huisarts of specialist	Opvolging	group 2
44	19	resultaten van uitgevoerde onderzoeken chronologisch en zeer beknopt in bullet points opgelijst, duidelijk gescheiden van andere info	Huidige problematiek	group 2
45	20	Huidige medicatie voor de huidige ziekte. Kort en zakelijk. Het me est mogelijk onder vorm van bullets en labels.	Medicatie	group 2
46	21	Eventueel standaardprocedure voor structuur overkoepelend. Liefst eenvoudige structuur	Structuur/vorm	group 2
47	22	is diagnose besproken met patiënt of niet (ja/nee) - beknopt	Andere / Ik weet het niet	group 2

48	23	mogelijke diagnoses die nog worden uitgewerkt opgelijst op einde brief	Opvolging	group 2
49	24	Consent/ patient akkoord met voorgesteld beleid	Communicatie	group 2
50	25	Duidelijke en aparte vermelding allergieen	Andere / Ik weet het niet	group 2
51	26	Duidelijk beleid ivm ontslag, opname rvt, andere	Opvolging	group 2
52	27	In alinea's	Structuur/vorm	group 2
53	28	Toekomstige afspraken	Opvolging	group 2
54	29	aandachtspunten naar huisarts bij opvolging - alarmtekenen voor verwijzing/actie	Opvolging	group 2
55	1	Voorgeschiedenis in bullets	Voorgeschiedenis	group 3
56	2	Voorgeschiedenis chronologisch	Voorgeschiedenis	group 3
57	3	Medicatiewijziging duidelijk aanwezig	Medicatie	group 3
58	4	Voorgeschiedenis opdelen in thema: chirurgisch, immuniteit die al eens getest is (vb EBV IgM)	Voorgeschiedenis	group 3
59	5	To do lijst bij ontslag	Opvolging	group 3
60	6	Aanmeldreden en Hoofddiagnose opname	Huidige problematiek	group 3
61	7	Zorgplanning afspraken	Structuur/vorm	group 3
62	8	Chirurgische voorgeschiedenis (enkel buik operaties)	Voorgeschiedenis	group 3
63	9	Voorgeschiedenis: kort overzicht stollingsrisico zoals roken, bmi, DVT	Voorgeschiedenis	group 3
64	10	max 1 A4 blad van de totale tekst	Structuur/vorm	group 3
65	11	Specifieke Allergie duidelijk vermeld bovenaan samenvatting met extra aandachtig voor levensbedreigende zaken	Voorgeschiedenis	group 3
66	12	Laatste terugbetaalde PAP afname incl resultaat	Voorgeschiedenis	group 3
67	13	nieuwe opgestarte medicatie in besluit	Besluit	group 3
68	14	allergie: Latex en penicilline duidelijk vermeld incl soort reactie	Voorgeschiedenis	group 3
69	15	Medicatie : duidelijke weergave van bloedverdunners	Voorgeschiedenis	group 3
70	16	onderzoeken die nog dienen te gebeuren onderaan in besluit	Besluit	group 3
71	17	Kort en bondig opnameverloop in 2 korte zinnen	Structuur/vorm	group 3
72	18	Medicatie wijziging, hoelang en wanneer te nemen, nodige follow up (vb wanneer labo nodig)	Medicatie	group 3
73	19	Relevante operatieve voorgeschiedenis chronologisch en in een aparte categorie met de meest relevante medische voorgeschiedenis. Levensbedreigende allergieën in het vet geschreven.	Voorgeschiedenis	group 3
74	20	reden van consultatie of opname in aparte heading	Huidige problematiek	group 3

75	21	Beeldvorming welke tijdens opname gebeurde - met kort besluit	Huidige problematiek	group 3
76	22	besluit in bullets, max 10 bullets, in korte zinnnen en geordend per orgaanproblematiek	Besluit	group 3
77	23	Samenvatting moet snel kunnen gelezen worden	Structuur/vorm	group 3
78	24	medicatie apart, maar enkel wat aangepast is sinds deze opname of consultatie: nieuw, gestopt, veranderd	Medicatie	group 3
79	25	Obstetrische voorgeschienis GPA en chronologische weergave met vermelding termijn van partus, modus partus en zwangerschapscomplicaties	Voorgeschiedenis	group 3
80	26	opnameverloop niet includeren	Structuur/vorm	group 3
81	27	Reden opname. Diagnose bij ontslag/tijdens opname. Eventueel uitgevoerde ingrepen in het vet. Indien geen operatie diagnose in het vet.	Huidige problematiek	group 3
82	28	Vermelding inclusie diabeteszorgtraject / vermelding inclusie nierinsufficientiezorgtrajcet	Opvolging	group 3
83	29	Bij nieuw opgestarte medicatie, eventueel mogelijke alternatieven indien doel niet gehaald, of bijwerkingen	Medicatie	group 3
84	30	diagnoses of procedures uit de voorgeschiedenis niet in besluit	Besluit	group 3
85	31	Geen nutteloze technisch onderzoeken vermelden zowel labo en medische beeldvorming	Structuur/vorm	group 3
86	32	geen laboparameters in ontslagbrief	Andere / Ik weet het niet	group 3
87	33	Chirurgisch verslag: duidelijke weergave of er vreemd materiaal aanwezig is in het abdomen	Huidige problematiek	group 3
88	34	Medicatie in stofnaam	Medicatie	group 3
89	35	medicatie schematisch, met dat wat aangepast is in het vet gezet	Medicatie	group 3
90	36	Enkel wijzigingen van chronische medicatie. En duidelijk overzicht van nieuwe tijdelijke medicatie bij ontslag	Medicatie	group 3
91	37	DNR in opvolging, zorgplanning	Opvolging	group 3
92	38	controle afspraken in todo lijst	Opvolging	group 3
93	39	ISBAR reden van opname, background assssment, recommendations	Structuur/vorm	group 3
94	40	starten met arts en discipline	Structuur/vorm	group 3
95	41	geen operatieverslag	Structuur/vorm	group 3
96	42	Duidelijk en kort opsommen wie de opvolging zal doen, en wat er moet gebeuren.	Opvolging	group 3
97	43	medicatieschema in tabel met kleurcodes	Medicatie	group 3
98	44	verschil tss chronische en tijdelijke medicatie	Medicatie	group 3
99	45	dnr planning	Communicatie	group 3
100	46	Duidelijk vermelden van zorg afspraken en palliatief statuut	Communicatie	group 3
101	47	Visueel werken met tabellen, woorden in het vet, kleuren, interlinie	Structuur/vorm	group 3
102	48	kerndiagnoses en procedures in bold (maar niet in voorgeschiedenis)	Structuur/vorm	group 3

103	49	Gebruik van korte zinnen met kernwoorden in het vet	Structuur/vorm	group 3
104	50	Belangrijkste boodschap voor patiënt/ voor familie/ voor huisarts meegeven. Indien relevant.	Communicatie	group 3
105	51	overlijden patient moet bovenaan de brief staan, met kruisje vooraan de zin	Andere / Ik weet het niet	group 3
106	1	Chronologisch	Structuur/vorm	group 4
107	2	Op jaartal	Structuur/vorm	group 4
108	3	Voorgeschiedenis vetgedrukt indien relevant voor huidige problematiek	Structuur/vorm	group 4
109	4	Besluit in bullet points	Structuur/vorm	group 4
110	5	Bovenaan de brief	Besluit	group 4
111	6	Cf. abstract van een wetenschappelijk artikel	Besluit	group 4
112	7	Afkortingen eerste maal voluit en nadien (afkorting)	Structuur/vorm	group 4
113	8	Medicatielijst bij opname	Medicatie	group 4
114	9	Bij besluit: enkel aangepaste medicatie	Medicatie	group 4
115	10	Planning voor de patiënt	Opvolging	group 4
116	11	Synthese verantwoordelijkheden in bullet points. (vb. huisarts doet x-y-z, specialist a doet b-c-d)	Opvolging	group 4
117	12	Automatisch gegenereerde links naar extra informatie bij minder gekende pathologie/operaties met aangepaste anatomie/...	Andere / Ik weet het niet	group 4
118	13	Beknopte voorgeschiedenis op geleide van functieprofiel zorgverlener, zorgvraag patiënt, etc.	Voorgeschiedenis	group 4
119	14	Lijst uitgevoerde onderzoeken (zonder besluit)	Andere / Ik weet het niet	group 4
120	15	Reden van opname	Huidige problematiek	group 4
121	16	Uniform taalgebruik voor eenzelfde problematiek	Structuur/vorm	group 4
122	17	Vroegtijdige zorgplanning (geÃ¯nitieerd, eventuele aanpassingen, etc.)	Opvolging	group 4
123	18	Hoofdbehandelaar + contactgegevens.	Opvolging	group 4
124	19	Enkel wat consequenties heeft voor behandeling patiënt behouden. Overbodige elementen i.k.v. bv. MKG registratie niet weergeven.	Besluit	group 4
125	20	Aparte alinea per onderdeel.	Structuur/vorm	group 4
126	21	Psychosociale context van de patiënt	Communicatie	group 4
127	1	Automatisch aanvullen Medische voorgeschiedenis	Voorgeschiedenis	group 5
128	2	Beknopte voorgeschiedenis in bulletpoints chronologish	Voorgeschiedenis	group 5

129	3	Chronologisch, voorafgegaan van datum<br><br>Aangevuld met nieuwe/huidige diagnose<br><br>Opgesplitst in relevante, niet relevante en therapeutische voorgeschiedenis<br><br>Opgesplitst in hoofd- en bijdiagnose<br><br>Elk item gekoppeld aan een ICPC-code<br><br>Filter wat niet met voorgeschiedenis te maken eruit.	Voorgeschiedenis	group 5
130	4	Medicatielijst gestandardiseerd	Medicatie	group 5
131	5	ontslagmedicatie	Medicatie	group 5
132	6	Voorgeschiedenis	Voorgeschiedenis	group 5
133	7	Reden van opname	Structuur/vorm	group 5
134	8	Medicatie aanpassingen gecategoriseerd volgens gestopt, opgestart, gewijzigd	Medicatie	group 5
135	9	Beloop van de opname	Opvolging	group 5
136	10	Diagnose, interventie, opmerking<br><br>Zo nog DD of diagnostisch proces, planning en opvolging in standaard outline	Besluit	group 5
137	11	Reden van opname	Huidige problematiek	group 5
138	12	DNR code vermelden	Communicatie	group 5
139	13	technische onderzoeken	Andere / Ik weet het niet	group 5
140	14	Besluit	Besluit	group 5
141	15	Geplande afspraken	Opvolging	group 5
142	16	Opnameduur	Andere / Ik weet het niet	group 5
143	17	Ontslagdatum	Andere / Ik weet het niet	group 5

144	18	In tabelvorm<br><br>Met de actieve stof vermeld<br><br>Opgesplitst volgens chronisch acuut<br><br>Opgestarte medicatie gehighlight<br><br>Aangepaste op een andere manier gemarkeerd<br><br>Gestopte medicatie doorgehaald	Medicatie	group 5
145	19	Uitgevoerde onderzoeken beknopt in bulletpoints	Huidige problematiek	group 5
146	20	keuze tussen proza of bulletstijl (of kunen teruggrijpen naar proza)	Structuur/vorm	group 5
147	21	Type brief (metadata)Opvolging / consultatie<br>Opname<br>Intake/ nieuwe patiënt<br>Interventie / behandeling<br>Discipline	Structuur/vorm	group 5
148	22	Voor (MKA) chirurgie relevante medicatie: botombouwremmers (bisfosfonaten) en ontstollende medicatie	Medicatie	group 5
149	23	Hoofddiagnose, nevendiagnose	Andere / Ik weet het niet	group 5
150	24	praktische opvolging van pt (bij zeldzame diagnoses)	Opvolging	group 5
151	25	Opvolging door de huisarts	Opvolging	group 5
152	26	In tekstvorm<br><br>De samenvatting / besluit van de brief	Besluit	group 5
153	27	Beknopte conclusie	Besluit	group 5
154	28	heading voor multidisciplanaire zorg (sociale opvolging, thuishulp, partners...)	Communicatie	group 5
155	29	Belangrijkste info uit de anamnese<br><br>Samenvatting van de uitgevoerde onderzoeken	Huidige problematiek	group 5
156	30	links (cozo) naar onderzoeken in besluit zo mogelijk?	Structuur/vorm	group 5

		In tabelvorm<br><br>Welke therapieÃ«n werden opgestart<br><br>Wat moet er nog opgestart worden<br><br>Planning, welke consulten, welke disciplines<br><br>Welke opvolging is nog gepland		
157	31	Toevallige vondsten/ blinde vlekken	Opvolging	group 5
158	32	interactieve view	Structuur/vorm	group 5
159	33	Transfer van afdelingen	Huidige problematiek	group 5
160	34	labellen van "gevaarlijke" medicatie (bijwerkingen, interacties, ...)	Medicatie	group 5
161	35	Voorgeschiedenis per orgaansysteem kunnen organiseren	Voorgeschiedenis	group 5
162	36	Geen labo waarden	Structuur/vorm	group 5
163	37	Geen protocol beeldvorming	Structuur/vorm	group 5
164	38	In bullet points de uitgevoerde onderzoeken<br><br>Detail van het onderzoek uitklapbaar	Huidige problematiek	group 5
165	39	Nieuwe diagnoses	Besluit	group 5
166	40	Voor psychiatrische patiënt, vorige ingenomen psychofarmaca en klinisch effect of criteria voor stoppen	Andere / Ik weet het niet	group 5
167	41	Huidige problematiek / reden van opname eerst: kortBesluit: proza Verdere planning / afspraken / opvolgingVoorgeschiedenis: puntenMedicatie bij ontslag: punten, aanduiding van de wijzigingen Huidige anamnese Verloop van de opname: proza?Technische onderzoeken: punten, link naar het volledige resultaat	Structuur/vorm	group 5
168	42	Eventuele opnameduur<br><br>Eventuele ontslagdatum	Besluit	group 5
169	43	ICD10 klassificatie vermelden	Besluit	group 5
170	44	Betrokken disciplines<br>Welke info kreeg patiënt<br>DNR<br>Informeer consent<br>Alarmsignalen aan patiënt<br>Akkoord patiënt voor behandeling	Communicatie	group 5

Appendix H – Questionnaire

H.1 Table S7 Overview questionnaire (Dutch)

number	question_label	anwers_options	jump_to_question
0	Kun je de volgorde aangeven voor de secties die je in je ontslagbrief wil	['Algemeen', 'Voorgeschiedenis', 'Medicatie', 'Onderzoeken', 'Opnameverloop', 'Follow-up', 'Besluit']	[1, 1, 1, 1, 1, 1, 1]
1	Wil je sectie Algemeen in je samenvatting zien?	['Ja', 'Nee']	[2, 42]
2	Wil je Ligduur in de sectie Algemeen zien?	['Ja', 'Nee']	[3, 5]
3	Waar wil je dit zien?	['Onderaan', 'Bovenaan', 'Nieuwe Alinea']	[4, 4, 4]
4	Welke opmaak moet gebruikt worden?	['Geen', 'Onderlijnd', 'Italic', 'Vet', 'Doorgehaald', 'Fluo markering']	[5, 5, 5, 5, 5, 5]
5	Wil je Opnamedatum in de sectie Algemeen zien?	['Ja', 'Nee']	[6, 8]
6	Waar wil je dit zien?	['Onderaan', 'Bovenaan', 'Nieuwe Alinea']	[7, 7, 7]
7	Welke opmaak moet gebruikt worden?	['Geen', 'Onderlijnd', 'Italic', 'Vet', 'Doorgehaald', 'Fluo markering']	[8, 8, 8, 8, 8, 8]
8	Wil je Ontslagdatum in de sectie Algemeen zien?	['Ja', 'Nee']	[9, 11]
9	Waar wil je dit zien?	['Onderaan', 'Bovenaan', 'Nieuwe Alinea']	[10, 10, 10]
10	Welke opmaak moet gebruikt worden?	['Geen', 'Onderlijnd', 'Italic', 'Vet', 'Doorgehaald', 'Fluo markering']	[11, 11, 11, 11, 11, 11]
11	Wil je Reden van opname in de sectie Algemeen zien?	['Ja', 'Nee']	[12, 14]
12	Waar wil je dit zien?	['Onderaan', 'Bovenaan', 'Nieuwe Alinea']	[13, 13, 13]
13	Welke opmaak moet gebruikt worden?	['Geen', 'Onderlijnd', 'Italic', 'Vet', 'Doorgehaald', 'Fluo markering']	[14, 14, 14, 14, 14, 14]
14	Wil je Type brief (ontslagbrief, consultatiebrief) in de sectie Algemeen zien?	['Ja', 'Nee']	[15, 17]
15	Waar wil je dit zien?	['Onderaan', 'Bovenaan', 'Nieuwe Alinea']	[16, 16, 16]
16	Welke opmaak moet gebruikt worden?	['Geen', 'Onderlijnd', 'Italic', 'Vet', 'Doorgehaald', 'Fluo markering']	[17, 17, 17, 17, 17, 17]
17	Wil je Nieuwe patient in de sectie Algemeen zien?	['Ja', 'Nee']	[18, 20]
18	Waar wil je dit zien?	['Onderaan', 'Bovenaan', 'Nieuwe Alinea']	[19, 19, 19]
19	Welke opmaak moet gebruikt worden?	['Geen', 'Onderlijnd', 'Italic', 'Vet', 'Doorgehaald', 'Fluo markering']	[20, 20, 20, 20, 20, 20]
20	Wil je Discipline in de sectie Algemeen zien?	['Ja', 'Nee']	[21, 23]

21	Waar wil je dit zien?	['Onderaan', 'Bovenaan', 'Nieuwe Alinea']	[22, 22, 22]
22	Welke opmaak moet gebruikt worden?	['Geen', 'Onderlijnd', 'Italic', 'Vet', 'Doorgehaald', 'Fluo markering']	[23, 23, 23, 23, 23, 23]
23	Wil je Naam en gegevens behandelende arts in de sectie Algemeen zien?	['Ja', 'Nee']	[24, 26]
24	Waar wil je dit zien?	['Onderaan', 'Bovenaan', 'Nieuwe Alinea']	[25, 25, 25]
25	Welke opmaak moet gebruikt worden?	['Geen', 'Onderlijnd', 'Italic', 'Vet', 'Doorgehaald', 'Fluo markering']	[26, 26, 26, 26, 26, 26]
26	Wil je Opnamedienst in de sectie Algemeen zien?	['Ja', 'Nee']	[27, 29]
27	Waar wil je dit zien?	['Onderaan', 'Bovenaan', 'Nieuwe Alinea']	[28, 28, 28]
28	Welke opmaak moet gebruikt worden?	['Geen', 'Onderlijnd', 'Italic', 'Vet', 'Doorgehaald', 'Fluo markering']	[29, 29, 29, 29, 29, 29]
29	Wil je Bestemming in de sectie Algemeen zien?	['Ja', 'Nee']	[30, 32]
30	Waar wil je dit zien?	['Onderaan', 'Bovenaan', 'Nieuwe Alinea']	[31, 31, 31]
31	Welke opmaak moet gebruikt worden?	['Geen', 'Onderlijnd', 'Italic', 'Vet', 'Doorgehaald', 'Fluo markering']	[32, 32, 32, 32, 32, 32]
32	Wil je Palliatief in de sectie Algemeen zien?	['Ja', 'Nee']	[33, 35]
33	Waar wil je dit zien?	['Onderaan', 'Bovenaan', 'Nieuwe Alinea']	[34, 34, 34]
34	Welke opmaak moet gebruikt worden?	['Geen', 'Onderlijnd', 'Italic', 'Vet', 'Doorgehaald', 'Fluo markering']	[35, 35, 35, 35, 35, 35]
35	Wil je DNR in de sectie Algemeen zien?	['Ja', 'Nee']	[36, 38]
36	Waar wil je dit zien?	['Onderaan', 'Bovenaan', 'Nieuwe Alinea']	[37, 37, 37]
37	Welke opmaak moet gebruikt worden?	['Geen', 'Onderlijnd', 'Italic', 'Vet', 'Doorgehaald', 'Fluo markering']	[38, 38, 38, 38, 38, 38]
38	Wil je Overleden in de sectie Algemeen zien?	['Ja', 'Nee']	[39, 39]
39	Wil je Psychosociale context in de sectie Algemeen zien?	['Ja', 'Nee']	[40, 42]
40	Waar wil je dit zien?	['Onderaan', 'Bovenaan', 'Nieuwe Alinea']	[41, 41, 41]
41	Welke opmaak moet gebruikt worden?	['Geen', 'Onderlijnd', 'Italic', 'Vet', 'Doorgehaald', 'Fluo markering']	[42, 42, 42, 42, 42, 42]
42	Wil je sectie Voorgeschiedenis in je samenvatting zien?	['Ja', 'Nee']	[43, 113]
43	Wil je Volledige voorgeschiedenis in de sectie Voorgeschiedenis zien?	['Ja', 'Nee']	[44, 50]
44	Wil je ICD codes bij de Volledige voorgeschiedenis zien?	['Ja', 'Nee']	[45, 47]
45	Welke oplijsting wil je zien?	['Tabel', 'Bulletpoints - alle', 'Bulletpoints - < 10', 'Proza - korte kernwoorden', 'Proza - Volzinnen']	[46, 46, 46, 46, 46]
46	Welke opmaak moet gebruikt worden?	['Geen', 'Onderlijnd', 'Italic', 'Vet', 'Doorgehaald', 'Fluo markering']	[47, 47, 47, 47, 47, 47]
47	Wil je ICPC code bij de Volledige voorgeschiedenis zien?	['Ja', 'Nee']	[48, 50]
48	Welke oplijsting wil je zien?	['Tabel', 'Bulletpoints - alle', 'Bulletpoints - < 10', 'Proza - korte kernwoorden', 'Proza - Volzinnen']	[49, 49, 49, 49, 49]
49	Welke opmaak moet gebruikt worden?	['Geen', 'Onderlijnd', 'Italic', 'Vet', 'Doorgehaald', 'Fluo markering']	[50, 50, 50, 50, 50, 50]

50	Wil je Chirurgische Voorgeschiedenis in de sectie Voorgeschiedenis zien?	['Ja', 'Nee']	[51, 53]
51	Welke oplijsting wil je zien?	['Tabel', 'Bulletpoints - alle', 'Bulletpoints - < 10', 'Proza - korte kernwoorden', 'Proza - Volzinnen']	[52, 52, 52, 52, 52]
52	Welke opmaak moet gebruikt worden?	['Geen', 'Onderlijnd', 'Italic', 'Vet', 'Doorgehaald', 'Fluo markering']	[53, 53, 53, 53, 53, 53]
53	Wil je Per orgaansysteem in de sectie Voorgeschiedenis zien?	['Ja', 'Nee']	[54, 56]
54	Welke oplijsting wil je zien?	['Tabel', 'Bulletpoints - alle', 'Bulletpoints - < 10', 'Proza - korte kernwoorden', 'Proza - Volzinnen']	[55, 55, 55, 55, 55]
55	Welke opmaak moet gebruikt worden?	['Geen', 'Onderlijnd', 'Italic', 'Vet', 'Doorgehaald', 'Fluo markering']	[56, 56, 56, 56, 56, 56]
56	Wil je Gebruik tabak in de sectie Voorgeschiedenis zien?	['Ja', 'Nee']	[57, 59]
57	Waar wil je dit zien?	['Onderaan', 'Bovenaan', 'Nieuwe Alinea']	[58, 58, 58]
58	Welke opmaak moet gebruikt worden?	['Geen', 'Onderlijnd', 'Italic', 'Vet', 'Doorgehaald', 'Fluo markering']	[59, 59, 59, 59, 59, 59]
59	Wil je Geteste immuniteiten in de sectie Voorgeschiedenis zien?	['Ja', 'Nee']	[60, 62]
60	Welke oplijsting wil je zien?	['Tabel', 'Bulletpoints - alle', 'Bulletpoints - < 10', 'Proza - korte kernwoorden', 'Proza - Volzinnen']	[61, 61, 61, 61, 61]
61	Welke opmaak moet gebruikt worden?	['Geen', 'Onderlijnd', 'Italic', 'Vet', 'Doorgehaald', 'Fluo markering']	[62, 62, 62, 62, 62, 62]
62	Wil je Gebruik alcohol in de sectie Voorgeschiedenis zien?	['Ja', 'Nee']	[63, 65]
63	Waar wil je dit zien?	['Onderaan', 'Bovenaan', 'Nieuwe Alinea']	[64, 64, 64]
64	Welke opmaak moet gebruikt worden?	['Geen', 'Onderlijnd', 'Italic', 'Vet', 'Doorgehaald', 'Fluo markering']	[65, 65, 65, 65, 65, 65]
65	Wil je Nieuwe diagnosen in de sectie Voorgeschiedenis zien?	['Ja', 'Nee']	[66, 69]
66	Waar wil je dit zien?	['Onderaan', 'Bovenaan', 'Nieuwe Alinea']	[67, 67, 67]
67	Welke oplijsting wil je zien?	['Tabel', 'Bulletpoints - alle', 'Bulletpoints - < 10', 'Proza - korte kernwoorden', 'Proza - Volzinnen']	[68, 68, 68, 68, 68]
68	Welke opmaak moet gebruikt worden?	['Geen', 'Onderlijnd', 'Italic', 'Vet', 'Doorgehaald', 'Fluo markering']	[69, 69, 69, 69, 69, 69]
69	Wil je BMI in de sectie Voorgeschiedenis zien?	['Ja', 'Nee']	[70, 71]
70	Welke opmaak moet gebruikt worden?	['Geen', 'Onderlijnd', 'Italic', 'Vet', 'Doorgehaald', 'Fluo markering']	[71, 71, 71, 71, 71, 71]
71	Wil je Stollingsrisico in de sectie Voorgeschiedenis zien?	['Ja', 'Nee']	[72, 73]
72	Welke opmaak moet gebruikt worden?	['Geen', 'Onderlijnd', 'Italic', 'Vet', 'Doorgehaald', 'Fluo markering']	[73, 73, 73, 73, 73, 73]
73	Wil je Diep Veneuze Tromboze in de sectie Voorgeschiedenis zien?	['Ja', 'Nee']	[74, 75]
74	Welke opmaak moet gebruikt worden?	['Geen', 'Onderlijnd', 'Italic', 'Vet', 'Doorgehaald', 'Fluo markering']	[75, 75, 75, 75, 75, 75]
75	Wil je Obstetrische Voorgeschiedenis in de sectie Voorgeschiedenis zien?	['Ja', 'Nee']	[76, 91]
76	Wil je GPA bij de Obstetrische Voorgeschiedenis zien?	['Ja', 'Nee']	[77, 79]
77	Welke oplijsting wil je zien?	['Tabel', 'Bulletpoints - alle', 'Bulletpoints - < 10', 'Proza - korte kernwoorden', 'Proza - Volzinnen']	[78, 78, 78, 78, 78]
78	Welke opmaak moet gebruikt worden?	['Geen', 'Onderlijnd', 'Italic', 'Vet', 'Doorgehaald', 'Fluo markering']	[79, 79, 79, 79, 79, 79]

79	Wil je Laatste terugbetaalde PAP bij de Obstetrische Voorgeschiedenis zien?	['Ja', 'Nee']	[80, 82]
80	Welke oplijsting wil je zien?	['Tabel', 'Bulletpoints - alle', 'Bulletpoints - < 10', 'Proza - korte kernwoorden', 'Proza - Volzinnen']	[81, 81, 81, 81, 81]
81	Welke opmaak moet gebruikt worden?	['Geen', 'Onderlijnd', 'Italic', 'Vet', 'Doorgehaald', 'Fluo markering']	[82, 82, 82, 82, 82, 82]
82	Wil je Termijn partus bij de Obstetrische Voorgeschiedenis zien?	['Ja', 'Nee']	[83, 85]
83	Welke oplijsting wil je zien?	['Tabel', 'Bulletpoints - alle', 'Bulletpoints - < 10', 'Proza - korte kernwoorden', 'Proza - Volzinnen']	[84, 84, 84, 84, 84]
84	Welke opmaak moet gebruikt worden?	['Geen', 'Onderlijnd', 'Italic', 'Vet', 'Doorgehaald', 'Fluo markering']	[85, 85, 85, 85, 85, 85]
85	Wil je Zwangerschapcomplicaties bij de Obstetrische Voorgeschiedenis zien?	['Ja', 'Nee']	[86, 88]
86	Welke oplijsting wil je zien?	['Tabel', 'Bulletpoints - alle', 'Bulletpoints - < 10', 'Proza - korte kernwoorden', 'Proza - Volzinnen']	[87, 87, 87, 87, 87]
87	Welke opmaak moet gebruikt worden?	['Geen', 'Onderlijnd', 'Italic', 'Vet', 'Doorgehaald', 'Fluo markering']	[88, 88, 88, 88, 88, 88]
88	Wil je Modus partus bij de Obstetrische Voorgeschiedenis zien?	['Ja', 'Nee']	[89, 91]
89	Welke oplijsting wil je zien?	['Tabel', 'Bulletpoints - alle', 'Bulletpoints - < 10', 'Proza - korte kernwoorden', 'Proza - Volzinnen']	[90, 90, 90, 90, 90]
90	Welke opmaak moet gebruikt worden?	['Geen', 'Onderlijnd', 'Italic', 'Vet', 'Doorgehaald', 'Fluo markering']	[91, 91, 91, 91, 91, 91]
91	Wil je Nieuwe diagnosen en chronische ziekten in de sectie Voorgeschiedenis zien?	['Ja', 'Nee']	[92, 94]
92	Welke oplijsting wil je zien?	['Tabel', 'Bulletpoints - alle', 'Bulletpoints - < 10', 'Proza - korte kernwoorden', 'Proza - Volzinnen']	[93, 93, 93, 93, 93]
93	Welke opmaak moet gebruikt worden?	['Geen', 'Onderlijnd', 'Italic', 'Vet', 'Doorgehaald', 'Fluo markering']	[94, 94, 94, 94, 94, 94]
94	Wil je AllergieÃ«n in de sectie Voorgeschiedenis zien?	['Ja', 'Nee']	[95, 113]
95	Wil je Alle bij de AllergieÃ«n zien?	['Ja', 'Nee']	[96, 98]
96	Welke oplijsting wil je zien?	['Tabel', 'Bulletpoints - alle', 'Bulletpoints - < 10', 'Proza - korte kernwoorden', 'Proza - Volzinnen']	[97, 97, 97, 97, 97]
97	Welke opmaak moet gebruikt worden?	['Geen', 'Onderlijnd', 'Italic', 'Vet', 'Doorgehaald', 'Fluo markering']	[98, 98, 98, 98, 98, 98]
98	Wil je Latex bij de AllergieÃ«n zien?	['Ja', 'Nee']	[99, 101]
99	Welke oplijsting wil je zien?	['Tabel', 'Bulletpoints - alle', 'Bulletpoints - < 10', 'Proza - korte kernwoorden', 'Proza - Volzinnen']	[100, 100, 100, 100, 100]
100	Welke opmaak moet gebruikt worden?	['Geen', 'Onderlijnd', 'Italic', 'Vet', 'Doorgehaald', 'Fluo markering']	[101, 101, 101, 101, 101, 101]
101	Wil je Penicilline bij de AllergieÃ«n zien?	['Ja', 'Nee']	[102, 104]
102	Welke oplijsting wil je zien?	['Tabel', 'Bulletpoints - alle', 'Bulletpoints - < 10', 'Proza - korte kernwoorden', 'Proza - Volzinnen']	[103, 103, 103, 103, 103]
103	Welke opmaak moet gebruikt worden?	['Geen', 'Onderlijnd', 'Italic', 'Vet', 'Doorgehaald', 'Fluo markering']	[104, 104, 104, 104, 104, 104]
104	Wil je Reacties bij de AllergieÃ«n zien?	['Ja', 'Nee']	[105, 107]
105	Welke oplijsting wil je zien?	['Tabel', 'Bulletpoints - alle', 'Bulletpoints - < 10', 'Proza - korte kernwoorden', 'Proza - Volzinnen']	[106, 106, 106, 106, 106]
106	Welke opmaak moet gebruikt worden?	['Geen', 'Onderlijnd', 'Italic', 'Vet', 'Doorgehaald', 'Fluo markering']	[107, 107, 107, 107, 107, 107]
107	Wil je Nieuwe bij de AllergieÃ«n zien?	['Ja', 'Nee']	[108, 110]

108	Welke oplijsting wil je zien?	['Tabel', 'Bulletpoints - alle', 'Bulletpoints - < 10', 'Proza - korte kernwoorden', 'Proza - Volzinnen']	[109, 109, 109, 109, 109]
109	Welke opmaak moet gebruikt worden?	['Geen', 'Onderlijnd', 'Italic', 'Vet', 'Doorgehaald', 'Fluo markering']	[110, 110, 110, 110, 110, 110]
110	Wil je Levensbedreigende bij de AllergieÃ«n zien?	['Ja', 'Nee']	[111, 113]
111	Welke oplijsting wil je zien?	['Tabel', 'Bulletpoints - alle', 'Bulletpoints - < 10', 'Proza - korte kernwoorden', 'Proza - Volzinnen']	[112, 112, 112, 112, 112]
112	Welke opmaak moet gebruikt worden?	['Geen', 'Onderlijnd', 'Italic', 'Vet', 'Doorgehaald', 'Fluo markering']	[113, 113, 113, 113, 113, 113]
113	Wil je sectie Medicatie in je samenvatting zien?	['Ja', 'Nee']	[114, 179]
114	Wil je Alle in de sectie Medicatie zien?	['Ja', 'Nee']	[115, 127]
115	Wil je Startdatum bij de Alle zien?	['Ja', 'Nee']	[116, 119]
116	Welke oplijsting wil je zien?	['Tabel', 'Bulletpoints - alle', 'Bulletpoints - < 10', 'Proza - korte kernwoorden', 'Proza - Volzinnen']	[117, 117, 117, 117, 117]
117	Welke volgorde wil je zien?	['Chronologisch', 'Thematisch', 'Kleurcodes', 'Geen', 'Alfabetisch']	[118, 118, 118, 118, 118]
118	Welke opmaak moet gebruikt worden?	['Geen', 'Onderlijnd', 'Italic', 'Vet', 'Doorgehaald', 'Fluo markering']	[119, 119, 119, 119, 119, 119]
119	Wil je Duur bij de Alle zien?	['Ja', 'Nee']	[120, 123]
120	Welke oplijsting wil je zien?	['Tabel', 'Bulletpoints - alle', 'Bulletpoints - < 10', 'Proza - korte kernwoorden', 'Proza - Volzinnen']	[121, 121, 121, 121, 121]
121	Welke volgorde wil je zien?	['Chronologisch', 'Thematisch', 'Kleurcodes', 'Geen', 'Alfabetisch']	[122, 122, 122, 122, 122]
122	Welke opmaak moet gebruikt worden?	['Geen', 'Onderlijnd', 'Italic', 'Vet', 'Doorgehaald', 'Fluo markering']	[123, 123, 123, 123, 123, 123]
123	Wil je Dosis bij de Alle zien?	['Ja', 'Nee']	[124, 127]
124	Welke oplijsting wil je zien?	['Tabel', 'Bulletpoints - alle', 'Bulletpoints - < 10', 'Proza - korte kernwoorden', 'Proza - Volzinnen']	[125, 125, 125, 125, 125]
125	Welke volgorde wil je zien?	['Chronologisch', 'Thematisch', 'Kleurcodes', 'Geen', 'Alfabetisch']	[126, 126, 126, 126, 126]
126	Welke opmaak moet gebruikt worden?	['Geen', 'Onderlijnd', 'Italic', 'Vet', 'Doorgehaald', 'Fluo markering']	[127, 127, 127, 127, 127, 127]
127	Wil je Gewijzigd in de sectie Medicatie zien?	['Ja', 'Nee']	[128, 140]
128	Wil je Startdatum bij de Gewijzigd zien?	['Ja', 'Nee']	[129, 132]
129	Welke volgorde wil je zien?	['Chronologisch', 'Thematisch', 'Kleurcodes', 'Geen', 'Alfabetisch']	[130, 130, 130, 130, 130]
130	Welke oplijsting wil je zien?	['Tabel', 'Bulletpoints - alle', 'Bulletpoints - < 10', 'Proza - korte kernwoorden', 'Proza - Volzinnen']	[131, 131, 131, 131, 131]
131	Welke opmaak moet gebruikt worden?	['Geen', 'Onderlijnd', 'Italic', 'Vet', 'Doorgehaald', 'Fluo markering']	[132, 132, 132, 132, 132, 132]
132	Wil je Duur bij de Gewijzigd zien?	['Ja', 'Nee']	[133, 136]
133	Welke volgorde wil je zien?	['Chronologisch', 'Thematisch', 'Kleurcodes', 'Geen', 'Alfabetisch']	[134, 134, 134, 134, 134]
134	Welke oplijsting wil je zien?	['Tabel', 'Bulletpoints - alle', 'Bulletpoints - < 10', 'Proza - korte kernwoorden', 'Proza - Volzinnen']	[135, 135, 135, 135, 135]
135	Welke opmaak moet gebruikt worden?	['Geen', 'Onderlijnd', 'Italic', 'Vet', 'Doorgehaald', 'Fluo markering']	[136, 136, 136, 136, 136, 136]
136	Wil je Dosis bij de Gewijzigd zien?	['Ja', 'Nee']	[137, 140]

137	Welke volgorde wil je zien?	['Chronologisch', 'Thematisch', 'Kleurcodes', 'Geen', 'Alfabetisch']	[138, 138, 138, 138, 138]
138	Welke oplijsting wil je zien?	['Tabel', 'Bulletpoints - alle', 'Bulletpoints - < 10', 'Proza - korte kernwoorden', 'Proza - Volzinnen']	[139, 139, 139, 139, 139]
139	Welke opmaak moet gebruikt worden?	['Geen', 'Onderlijnd', 'Italic', 'Vet', 'Doorgehaald', 'Fluo markering']	[140, 140, 140, 140, 140, 140]
140	Wil je Gestopt in de sectie Medicatie zien?	['Ja', 'Nee']	[141, 141]
141	Wil je Gestart / Nieuwe in de sectie Medicatie zien?	['Ja', 'Nee']	[142, 145]
142	Welke volgorde wil je zien?	['Chronologisch', 'Thematisch', 'Kleurcodes', 'Geen', 'Alfabetisch']	[143, 143, 143, 143, 143]
143	Welke oplijsting wil je zien?	['Tabel', 'Bulletpoints - alle', 'Bulletpoints - < 10', 'Proza - korte kernwoorden', 'Proza - Volzinnen']	[144, 144, 144, 144, 144]
144	Welke opmaak moet gebruikt worden?	['Geen', 'Onderlijnd', 'Italic', 'Vet', 'Doorgehaald', 'Fluo markering']	[145, 145, 145, 145, 145, 145]
145	Wil je Bij opname in de sectie Medicatie zien?	['Ja', 'Nee']	[146, 149]
146	Welke volgorde wil je zien?	['Chronologisch', 'Thematisch', 'Kleurcodes', 'Geen', 'Alfabetisch']	[147, 147, 147, 147, 147]
147	Welke oplijsting wil je zien?	['Tabel', 'Bulletpoints - alle', 'Bulletpoints - < 10', 'Proza - korte kernwoorden', 'Proza - Volzinnen']	[148, 148, 148, 148, 148]
148	Welke opmaak moet gebruikt worden?	['Geen', 'Onderlijnd', 'Italic', 'Vet', 'Doorgehaald', 'Fluo markering']	[149, 149, 149, 149, 149, 149]
149	Wil je Bij ontslag in de sectie Medicatie zien?	['Ja', 'Nee']	[150, 153]
150	Welke volgorde wil je zien?	['Chronologisch', 'Thematisch', 'Kleurcodes', 'Geen', 'Alfabetisch']	[151, 151, 151, 151, 151]
151	Welke oplijsting wil je zien?	['Tabel', 'Bulletpoints - alle', 'Bulletpoints - < 10', 'Proza - korte kernwoorden', 'Proza - Volzinnen']	[152, 152, 152, 152, 152]
152	Welke opmaak moet gebruikt worden?	['Geen', 'Onderlijnd', 'Italic', 'Vet', 'Doorgehaald', 'Fluo markering']	[153, 153, 153, 153, 153, 153]
153	Wil je Aanduiding tijdelijk versus Chronisch in de sectie Medicatie zien?	['Ja', 'Nee']	[154, 156]
154	Welke oplijsting wil je zien?	['Tabel', 'Bulletpoints - alle', 'Bulletpoints - < 10', 'Proza - korte kernwoorden', 'Proza - Volzinnen']	[155, 155, 155, 155, 155]
155	Welke opmaak moet gebruikt worden?	['Geen', 'Onderlijnd', 'Italic', 'Vet', 'Doorgehaald', 'Fluo markering']	[156, 156, 156, 156, 156, 156]
156	Wil je Stofnaam weergeven in de sectie Medicatie zien?	['Ja', 'Nee']	[157, 158]
157	Welke oplijsting wil je zien?	['Tabel', 'Bulletpoints - alle', 'Bulletpoints - < 10', 'Proza - korte kernwoorden', 'Proza - Volzinnen']	[158, 158, 158, 158, 158]
158	Wil je Bloedverdunners in de sectie Medicatie zien?	['Ja', 'Nee']	[159, 162]
159	Welke volgorde wil je zien?	['Chronologisch', 'Thematisch', 'Kleurcodes', 'Geen', 'Alfabetisch']	[160, 160, 160, 160, 160]
160	Welke oplijsting wil je zien?	['Tabel', 'Bulletpoints - alle', 'Bulletpoints - < 10', 'Proza - korte kernwoorden', 'Proza - Volzinnen']	[161, 161, 161, 161, 161]
161	Welke opmaak moet gebruikt worden?	['Geen', 'Onderlijnd', 'Italic', 'Vet', 'Doorgehaald', 'Fluo markering']	[162, 162, 162, 162, 162, 162]
162	Wil je Chirurgische medicatie (bisfosfonaten, ontstollende) in de sectie Medicatie zien?	['Ja', 'Nee']	[163, 166]
163	Welke volgorde wil je zien?	['Chronologisch', 'Thematisch', 'Kleurcodes', 'Geen', 'Alfabetisch']	[164, 164, 164, 164, 164]
164	Welke oplijsting wil je zien?	['Tabel', 'Bulletpoints - alle', 'Bulletpoints - < 10', 'Proza - korte kernwoorden', 'Proza - Volzinnen']	[165, 165, 165, 165, 165]
165	Welke opmaak moet gebruikt worden?	['Geen', 'Onderlijnd', 'Italic', 'Vet', 'Doorgehaald', 'Fluo markering']	[166, 166, 166, 166, 166, 166]

166	Wil je Alternatieve medicatie in de sectie Medicatie zien?	['Ja', 'Nee']	[167, 169]
167	Welke oplijsting wil je zien?	['Tabel', 'Bulletpoints - alle', 'Bulletpoints - < 10', 'Proza - korte kernwoorden', 'Proza - Volzinnen']	[168, 168, 168, 168, 168]
168	Welke opmaak moet gebruikt worden?	['Geen', 'Onderlijnd', 'Italic', 'Vet', 'Doorgehaald', 'Fluo markering']	[169, 169, 169, 169, 169, 169]
169	Wil je Bijwerkingen / interacties in de sectie Medicatie zien?	['Ja', 'Nee']	[170, 172]
170	Welke oplijsting wil je zien?	['Tabel', 'Bulletpoints - alle', 'Bulletpoints - < 10', 'Proza - korte kernwoorden', 'Proza - Volzinnen']	[171, 171, 171, 171, 171]
171	Welke opmaak moet gebruikt worden?	['Geen', 'Onderlijnd', 'Italic', 'Vet', 'Doorgehaald', 'Fluo markering']	[172, 172, 172, 172, 172, 172]
172	Wil je Psychofarmaca in de sectie Medicatie zien?	['Ja', 'Nee']	[173, 179]
173	Wil je klinisch effect bij de Psychofarmaca zien?	['Ja', 'Nee']	[174, 176]
174	Welke oplijsting wil je zien?	['Tabel', 'Bulletpoints - alle', 'Bulletpoints - < 10', 'Proza - korte kernwoorden', 'Proza - Volzinnen']	[175, 175, 175, 175, 175]
175	Welke opmaak moet gebruikt worden?	['Geen', 'Onderlijnd', 'Italic', 'Vet', 'Doorgehaald', 'Fluo markering']	[176, 176, 176, 176, 176, 176]
176	Wil je stop criteria bij de Psychofarmaca zien?	['Ja', 'Nee']	[177, 179]
177	Welke oplijsting wil je zien?	['Tabel', 'Bulletpoints - alle', 'Bulletpoints - < 10', 'Proza - korte kernwoorden', 'Proza - Volzinnen']	[178, 178, 178, 178, 178]
178	Welke opmaak moet gebruikt worden?	['Geen', 'Onderlijnd', 'Italic', 'Vet', 'Doorgehaald', 'Fluo markering']	[179, 179, 179, 179, 179, 179]
179	Wil je sectie Onderzoeken in je samenvatting zien?	['Ja', 'Nee']	[180, 198]
180	Wil je Technische onderzoeken in de sectie Onderzoeken zien?	['Ja', 'Nee']	[181, 181]
181	Wil je Laboratorium resultaten in de sectie Onderzoeken zien?	['Ja', 'Nee']	[182, 185]
182	Welke oplijsting wil je zien?	['Tabel', 'Bulletpoints - alle', 'Bulletpoints - < 10', 'Proza - korte kernwoorden', 'Proza - Volzinnen']	[183, 183, 183, 183, 183]
183	Welke volgorde wil je zien?	['Chronologisch', 'Thematisch', 'Kleurcodes', 'Geen', 'Alfabetisch']	[184, 184, 184, 184, 184]
184	Hoe wil je de details zien?	['Geen', '(Hyper)links', 'Tabbladen', 'In tekst zelf']	[185, 185, 185, 185]
185	Wil je Medische beeldvorming in de sectie Onderzoeken zien?	['Ja', 'Nee']	[186, 192]
186	Wil je Alle bij de Medische beeldvorming zien?	['Ja', 'Nee']	[187, 189]
187	Hoe wil je het protocol zien?	['Ja', 'Nee']	[188, 188]
188	Hoe wil je de details zien?	['Geen', '(Hyper)links', 'Tabbladen', 'In tekst zelf']	[189, 189, 189, 189]
189	Wil je Tijdens opname bij de Medische beeldvorming zien?	['Ja', 'Nee']	[190, 192]
190	Hoe wil je het protocol zien?	['Ja', 'Nee']	[191, 191]
191	Hoe wil je de details zien?	['Geen', '(Hyper)links', 'Tabbladen', 'In tekst zelf']	[192, 192, 192, 192]
192	Wil je Pre-opname onderzoeken in de sectie Onderzoeken zien?	['Ja', 'Nee']	[193, 194]
193	Welke oplijsting wil je zien?	['Tabel', 'Bulletpoints - alle', 'Bulletpoints - < 10', 'Proza - korte kernwoorden', 'Proza - Volzinnen']	[194, 194, 194, 194, 194]
194	Wil je Klinisch onderzoek in de sectie Onderzoeken zien?	['Ja', 'Nee']	[195, 198]

195	Welke oplijsting wil je zien?	['Tabel', 'Bulletpoints - alle', 'Bulletpoints - < 10', 'Proza - korte kernwoorden', 'Proza - Volzinnen']	[196, 196, 196, 196, 196]
196	Welke volgorde wil je zien?	['Chronologisch', 'Thematisch', 'Kleurcodes', 'Geen', 'Alfabetisch']	[197, 197, 197, 197, 197]
197	Hoe wil je de details zien?	['Geen', '(Hyper)links', 'Tabbladen', 'In tekst zelf']	[198, 198, 198, 198]
198	Wil je sectie Opnameverloop in je samenvatting zien?	['Ja', 'Nee']	[199, 230]
199	Wil je Nieuwe diagnosen in de sectie Opnameverloop zien?	['Ja', 'Nee']	[200, 209]
200	Wil je ICD-10 codes bij de Nieuwe diagnosen zien?	['Ja', 'Nee']	[201, 203]
201	Welke oplijsting wil je zien?	['Tabel', 'Bulletpoints - alle', 'Bulletpoints - < 10', 'Proza - korte kernwoorden', 'Proza - Volzinnen']	[202, 202, 202, 202, 202]

H.2 Table S8 Questionnaire - number of questions by each section

Section	n	p
Basic questions	3	3%
General	6	5%
Medical History	28	25%
Medication	15	14%
Examinations	14	13%
Admission history	26	24%
Follow-up	12	11%
Decision	5	5%
Final question	1	1%

Appendix I – Results questionnaire

I.1 Figure S6 Type of Health Care Provider and Years of Experience

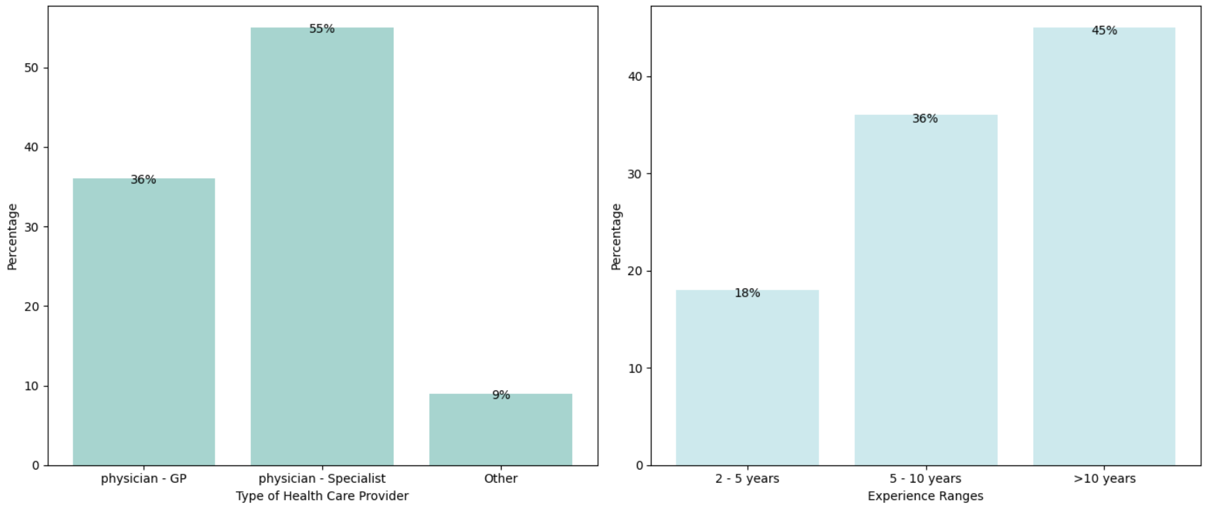

I.2 Figure S7 Percentage affirmative responses to whether participants wanted specific sections included in the summary

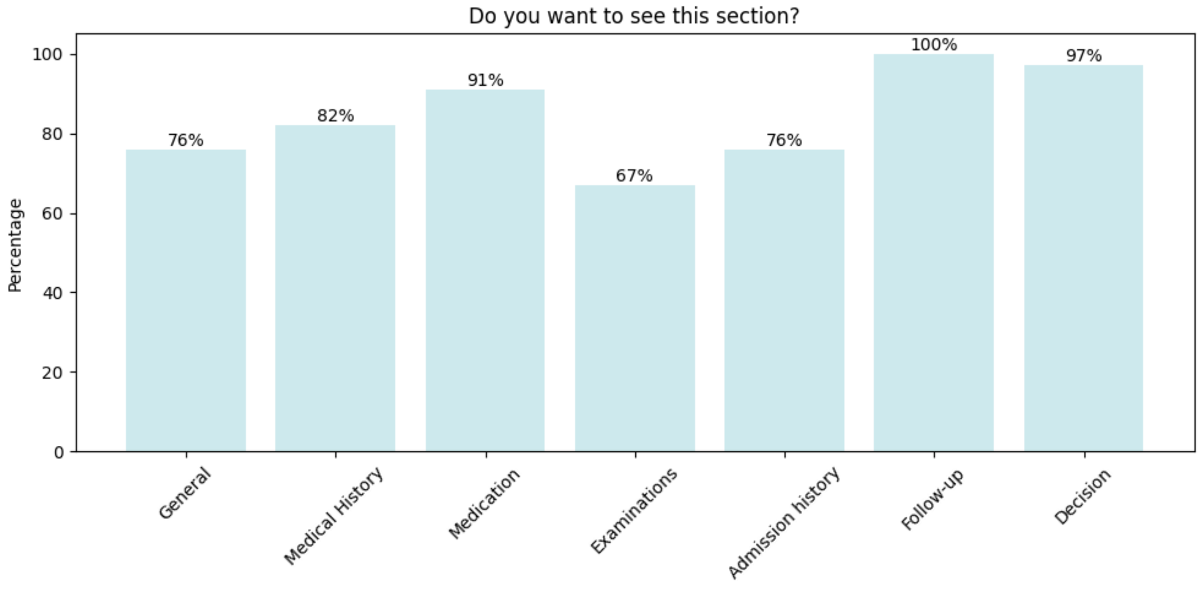

Appendix J – example individual prompt (English)

J.1 Figure S8 Example individual prompt (English)

```
This text is a medical discharge letter (the report of a hospital admission). I would like a
summary in Dutch.
    The intention is that I, as a healthcare provider, can use this letter to properly follow
up the patient.
    The summary must be a maximum of one page long and clearly structured.
    Use formal language based on the expertise of a doctor.
    Start with the patient's name, date of birth and the name of the hospital.
    All dates must be in the format day/month/year.
    If a requested part is not in the letter, bundle the missing parts at the bottom per
section in one sentence and use 'Not mentioned' as an indication.
    Each section must be clearly separate and in capital letters.I want the section in this
order: General - Medical History - Admission history - Decision - Medication - Follow-up -
Examinations;Show the General section in the summary;Show the recording date;Show the reason
for recording;Show the type of letter (letter of resignation, consultation letter);Show the
discipline;Show the dismissal date;Show the name and data treating doctor;Show the patient's
destination after dismissal;Show the palliative;Show the DNR;Show the deceased (indicate with
cross next to the name);Highlight the reason for recording;Highlight the discipline;Highlight
the DNR;Algemeen: Place the text in bold;Algemeen: Use short keywords;Show the section History
in the Summary;Show per organ problem;Voorgeschiedenis: Use short keywords;Show in section
History The use of tobacco;Gebruik van tabak: No specific formatting;Show in section History
The use of alcohol;Gebruik van alcohol: No specific formatting;Show in section History the
BMI;BMI: No specific formatting;Show in section History the tested immunities;Geteste
immuniteiten: Use bullet points;Geteste immuniteiten: No specific formatting;Show the
coagulation risk in section History Section;Stollingsrisico: No specific formatting;Show in
section History De Diep Venous Thrombosis;Diep Veneuze Trombose: No specific formatting;Show
the new diagnoses in section History;Nieuwe diagnosen: Place the text in italic;Show the
allergies in section History;Show the reaction on the allergy.;Show latex allergy;Show
penicillin allergy;Show life-threatening allergies;Allergieën: Use bullet points;Allergieën:
No specific formatting;Show the Medication section in the summary;Show the medication upon
dismissal;Show the changed medication (dose, frequency, shape);Show the stopped
medication;Show the started / new medication;Highlight the stopped medication;Highlight the
started / new medication;Medicatie: Place the text in italic;Show dosage of medication;Show
the frequency of the medication;Show substance name of medication;Show the criteria to stop or
adjust the medication;Show the reason to start, stop or adjust the medication;Medicatie: Place
this in table form;Arrange the items alfabetic;Highlight blood thinners;Highlight temporary
medication;Medicatie: Use a new paragraph.;Medicatie: Place the text in italic;Show the side
effects / interactions in section Medication;Bijwerkingen / interacties: Place the text in
italic;Show the Section Investigate in the Summary;Toon in section Investigate the clinical
investigation;Klinisch onderzoek: Use short keywords;Arrange the items thematically;Klinisch
onderzoek: Use (hyper)links to view the details.;Show in section Investigate the laboratory
results;Laboratoriumresultaten: Use a maximum of 10 bullet points;Arrange the items
chronologically;Laboratoriumresultaten: Use (hyper)links to view the details.;Toon in section
Investigate the medical imaging / technical investigations;Show the protocol;Medische
beeldvorming / Technische onderzoeken: Use (hyper)links to view the details.;Investigate show
in section Investigate the pre-admission examinations (e.g. ECG);Pre-opname onderzoeken (bijv.
ECG): Use short keywords;Show the Section Lock in the Summary;Show the main diagnosis in
section;Hoofddiagnose: Place the text in bold;Show the new diagnoses in section;Show per organ
problem;Nieuwe diagnosen: Use short keywords;Nieuwe diagnosen: Place the text in italic;Show
in section Acquisition of the interventions performed;Uitgevoerde ingrepen: No specific
formatting;Show in section Acquisition of the interventions performed;Uitgevoerde ingrepen:
Use short keywords;Arrange the items thematically;Uitgevoerde ingrepen: Use (hyper)links to
view the details.;Show in section Acquisition of the interventions performed;Uitgevoerde
ingrepen: Place this in table form;Arrange the items thematically;Uitgevoerde ingrepen: Use
(hyper)links to view the details.;Show the Follow-up section in the summary;Show the planned
consultations;Show the planned investigations;Show the follow -up appointments;Show the Todo
List (Wie-Wat-Waarder/ Points of attention per care provider);Show the multidisciplinary
follow -up (social, home help);Show the institutions involved;Show the inclusion in specific
care processes;Highlight the none;Follow-up: No specific formatting;Follow-up: Place this in
table form;Arrange the items chronologically;Show the Follow-up section in communication;Show
alarm signals for patient;Show message for GP;Show diagnosis discussed with
patient;Communicatie: No specific formatting;Show in the Follow-up section the psychosocial
context;Psychosociale context: Use short keywords;Psychosociale context: No specific
formatting;Show the section Decree in the summary;Show in section decides the ankle new items
```

(diagnoses, medication,);enkel nieuwe items (diagnosen, medicatie, …): Use short keywords;Besluit: Use short keywords;If a certain item is not available in the letter, bundle this by section, e.g. Allergie: MISSING;I want this letter to be a maximum of one page long and to be clearly structured.;It may be written in formal language based on the expertise of a physician.;Please start with the name of the patient, the date of birth, and the name of the hospital. All dates should be in the format day/month/year.;If a requested section is not in the letter, bundle the missing parts per section and use 'Not mentioned' as an indication.;Each section should be clearly separated and in capital letters.
We would like to provide some additional explanation:
The name of the sections should be translated like: General = Algemeen, Medical History = Voorgeschiedenis, Medication = Medicatie, Examinations = Onderzoeken, Admission history = Opnameverloop, Follow-up = Follow-up, Decision = Besluit.
If new diagnoses are requested in the history, this means that you add the new diagnoses that were made during admission to the history.
By interventions we mean operations.
The admission process describes the path that the patient takes during the admission, the evolution of the condition and the steps that are taken during the admission in chronological order. Here we also expect to report the administration of blood transfusions, radiotherapy or chemotherapy, the performance of diagnostic examinations such as biopsies, endoscopies, the placement of feeding tubes, etc.
In Medical imaging / Technical examinations we mean by the protocol all actions that are performed during this examination and all findings that are made in this.
If changes in medication are requested, we mean changes in dose, frequency and form and whether medication has been stopped completely or new medication has been started.
To do this, make a comparison between the medication on admission on the one hand and the medication on discharge and the medication at the therapy proposal on the other.
Medication that has remained the same is not mentioned in the case of changed medication. If the question is about the risk of clotting, mention blood clots/thrombosis and embolisms and the use of blood-thinning medication. Also mention here if you find anything about cerebral infarction/CVA or heart attack/AMIAMI. Clotting disorders should also be mentioned here.
Communication is about discussing the condition and the further policy with the patient and his family.
If a section should not be mentioned, leave it out of the summary completely.
The information in the summary should come from the original letter. An exception to this is if ICD codes or ICPC codes are mentioned.
When asking for ICD codes, the ICD-10 code should be mentioned next to the diagnosis, as can be found at https://icd10be.health.belgium.be/.
When asking for ICPC codes, you can find them at https://browser.icpc-3.info/.
If a patient has come from another hospital, mention this under general.
